# Sage, Rosemary, and Bay Laurel Hydrodistillation By-Products as a Source of Bioactive Compounds

**DOI:** 10.3390/plants12132394

**Published:** 2023-06-21

**Authors:** Anđela Miljanović, Maja Dent, Dorotea Grbin, Sandra Pedisić, Zoran Zorić, Zvonimir Marijanović, Igor Jerković, Ana Bielen

**Affiliations:** 1 Faculty of Food Technology and Biotechnology, University of Zagreb, Pierottijeva 6, 10 000 Zagreb, Croatia; andela.miljanovic@pbf.unizg.hr (A.M.); maja.dent@pbf.unizg.hr (M.D.); dorotea.polo@gmail.com (D.G.); sandra.pedisic@pbf.unizg.hr (S.P.); zoran.zoric@pbf.unizg.hr (Z.Z.); 2 Faculty of Chemistry and Technology, University of Split, Ruđera Boškovića 35, 21 000 Split, Croatia; zmarijanovic@ktf-split.hr (Z.M.); igor.jerkovic@ktf-split.hr (I.J.)

**Keywords:** hydrodistillation residues, hydrolate, Mediterranean wild plants, solid residue, water residue

## Abstract

Essential oils from Mediterranean wild plants are widely used, but the hydrodistillation residues produced in parallel with these essential oils are significantly understudied and underexploited. Since there are only fragmentary data in the literature, we have, for the first time, systematically analyzed the chemical composition of the by-products obtained after hydrodistillation of sage, bay laurel, and rosemary leaves, i.e., hydrolates, water residues, and solid residues. The chemical composition of the hydrolates changed compared to their respective essential oils towards the dominance of more hydrophilic, oxygenated compounds, such as camphor in sage, 1,8-cineole in bay laurel, and berbenone in rosemary. However, some compounds, mostly sesquiterpenes, which were present in considerable amounts in essential oils, were absent or only present in very small amounts in the hydrolates. Furthermore, both the water and the solid residues were rich in polyphenols, such as procyanidins in bay laurel and rosmarinic acid in rosemary and sage. In conclusion, we demonstrate the valuable chemical composition of sage, rosemary, and bay laurel hydrodistillation by-products and discuss a wide range of their possible applications.

## 1. Introduction

Due to their rich bioactive content, essential oils have a wide range of applications, and their international trade is increasing by an average of 10% annually [1]. They are used in numerous industries, such as fragrance and flavor, nutraceuticals, cosmetics, cosmeceuticals, aroma chemicals, aromatherapy, and pharmaceutical, with a total annual market value of >1000 billion dollars [2]. Essential oil quantity varies during the production process, depending on the extraction method and plant material used, usually yielding up to several mL per 100 g of the dry plant [3,4,5,6,7,8,9,10,11]. The increasing demand for essential oils has led to the development of many innovative methods to improve their yield and composition. Before hydrodistillation, plant material is often treated with ultrasound, microwaves, ohmic heating, or enzymes to disrupt the cell wall and improve the access of the solvent to the cell content, thus enhancing the release of bioactive compounds [3,4,8,10,12,13,14,15,16]. However, regardless of the production procedure, essential oils produce a significant quantity of by-products [17]. These potentially valuable hydrodistillation residues include three main fractions: hydrolate, water residue, and solid residue. Hydrolates generally retain dissolved components of essential oils and, consequently, a significant amount of volatile compounds [18,19,20,21,22], while solid and water residues are rich in polyphenols [8,23,24,25,26,27,28]. Despite their valuable content, these fractions are often underexploited or considered waste.

Hydrolates are aqueous aromatic solutions saturated with the water-soluble volatile compounds of essential oil. The volatile content of hydrolates is significantly lower, both in the number of compounds and in their concentration than in the respective essential oils, although it was shown that the concentration of some major, mostly oxygenated components can be higher in hydrolates [29,30,31,32]. Ratios of different groups of components differ between hydrolates and essential oils; for instance, the hydrolates contain a higher percentage of oxygenated monoterpenes and a lower percentage of monoterpene and sesquiterpenes hydrocarbons that are highly hydrophobic [33,34]. Hydrolates have applications in the food industry, the fragrant aroma industry, the cosmetic industry, and aromatherapy and represent the most commonly used by-product of essential oil production [20,21,22,35,36].

Other residues from hydrodistillation originate from the plant/water mixture that remains after hydrodistillation. This mixture can be filtered and used as two separate sources of polyphenols: liquid water residue [25,27,37] and the remaining plant material, i.e., the solid residue [23,24,26,27,28]. The plant material contains many hydrophilic compounds that remain dissolved in the water residue. For instance, water residue obtained after hydrodistillation of rosemary leaves and Taif rose was a colored solution rich in antioxidants [25,27]. It should be noted that water residues kept under ambient conditions have been shown to be prone to bacterial and fungal contamination, and, therefore, their release into the environment could lead to environmental risks [2].

The solid residues that remain after hydrodistillation can also be used as secondary raw material to obtain different bioactives using conventional extraction techniques, such as solvent extraction in an orbital shaker and Soxhlet apparatus, or innovative techniques, such as ultrasound bath, resulting in considerable concentrations of phenols and flavonoids in the final extracts [8,23,24,26,27]. When comparing different extraction procedures used to process solid hydrodistillation residues, ultrasound application is considered gentler compared to other extraction procedures, working at a lower temperature and for a shorter time, making it more suitable for the preservation of polyphenols from thermal degradation [38,39,40].

In this study, we focus on aromatic Mediterranean plants rich in bioactive compounds: rosemary *(Rosmarinus officinalis* L., Lamiaceae), sage (*Salvia officinalis* L., Lamiaceae), and bay laurel (*Laurus nobilis* L., Lauraceae) [8,13,41,42]. Their essential oils are frequently used, but hydrodistillation residues produced in parallel with these essential oils are much less explored. To date, there are no studies reporting on the chemical composition of water residues remaining after the hydrodistillation of sage and bay laurel leaves. At the same time, there are only a few reports that demonstrate the value composition of other hydrodistillation by-products of these plants: hydrolates of rosemary, sage, and bay laurel hydrolates rich in volatiles [35], rosemary and sage water residues rich in polyphenols [27], and solid residues remaining after hydrodistillation of bay laurel [8], rosemary [24,27,28] and sage [23,28] as a valuable secondary source of polyphenol components. Thus, further research on hydrodistillation by-products of Mediterranean aromatic plants is necessary. In this study, we have analyzed the chemical composition of the by-products obtained after the sage, bay laurel, and rosemary hydrodistillation procedures. Additionally, we have tested whether different hydrodistillation pre-treatments, i.e., reflux extraction, reflux extraction with the addition of cell wall-degrading enzymes, and ultrasound, affected the chemical composition of the hydrodistillation by-products.

## 2. Results and Discussion

### 2.1. Impact of Different Pre-Treatments on the Chemical Composition of Hydrodistillation By-Products

**Figure 1:**
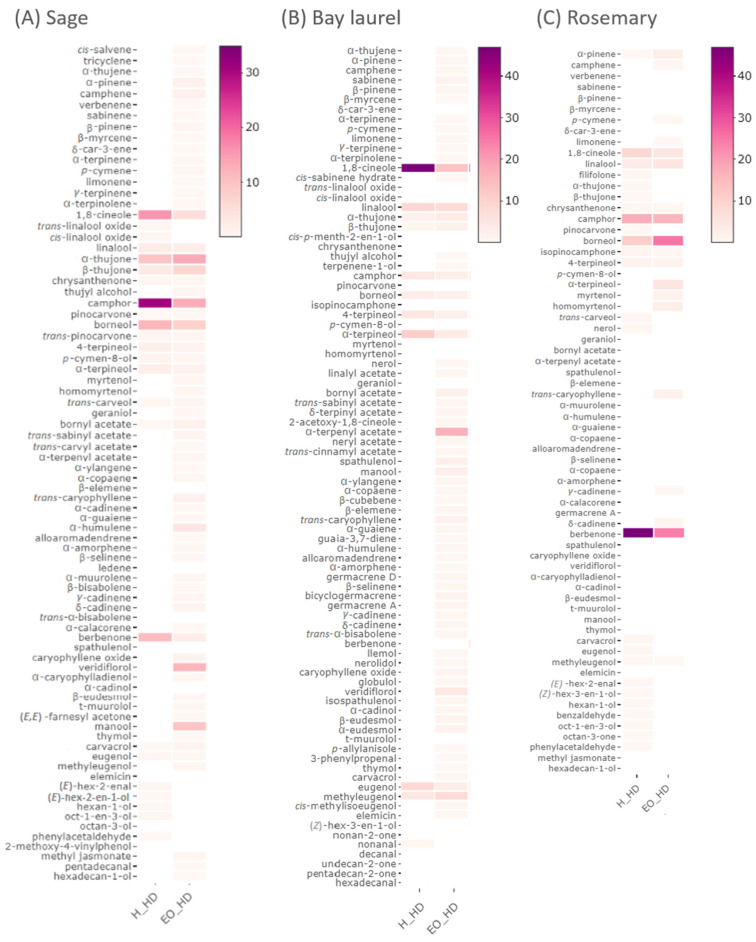
Heatmaps of the volatile content of sage, bay laurel, and rosemary hydrolates (obtained in this study) and their respective essential oils [45], as determined by GC-MS. Hydrolates (H_HD) and essential oils (EO_HD) were obtained by hydrodistillation without pre-treatments. Similar chemical profiles were obtained with different pre-treatments: HD-RE—hydrodistillation with reflux extraction pre-treatment, HD-US—hydrodistillation with ultrasound extraction pre-treatment, HD-REXPC—hydrodistillation with reflux extraction pre-treatment assisted with cell wall-degrading enzymes (xylanase, pectinase, and cellulase), and are shown in detail in Supplementary Tables S1–S3. The colors correspond to the percentage of a particular compound respective to the total peak area.

**Figure 2:**
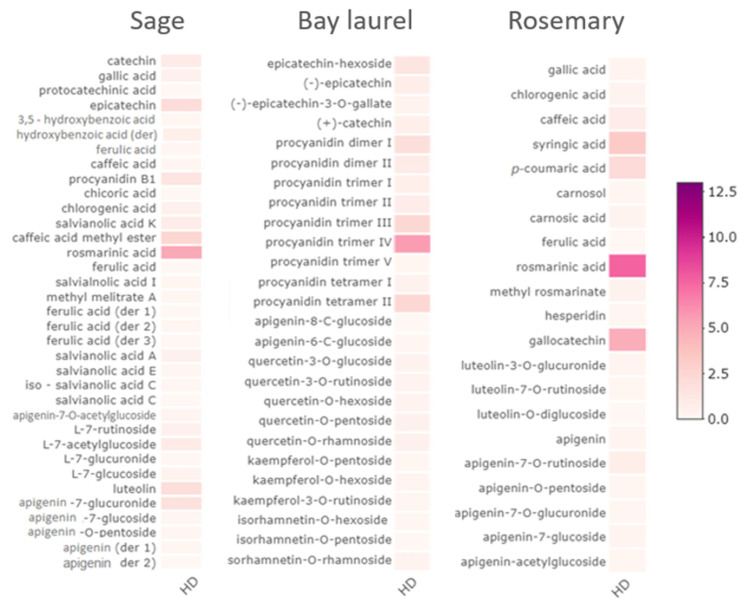
Heatmaps of the phenolic content of sage, bay laurel, and rosemary water residues, as determined by HPLC, after hydrodistillation (HD). Similar chemical profiles were obtained after different pre-treatments: HD-US—ultrasound extraction, HD-RE—reflux extraction, HD-REXPC—reflux extraction assisted with cell wall-degrading enzymes (xylanase, pectinase, and cellulase), and are shown in detail in Supplementary Tables S4–S6. The colors correspond to the mg of the compound per g of the dry plant. Authentic standards used for sage were (+)-catechin, (−)-epicatechin, apigenin, luteolin, rosmarinic acid, caffeic acid, gallic acid, ferulic acid, chicoric acid, chlorogenic acid, procyanidin B1, and protocatechinic acid; for bay laurel (+)-catechin, (−)-epicatechin, apigenin, quercetin-3-O-glucoside, quercetin-3-O-rutinoside, kaempferol-3-O-rutinoside, and for rosemary gallic acid, chlorogenic acid, caffeic acid, syringic acid, *p*-coumaric acid, ferulic acid, rosmarinic acid, apigenin, and luteolin.

**Figure 3:**
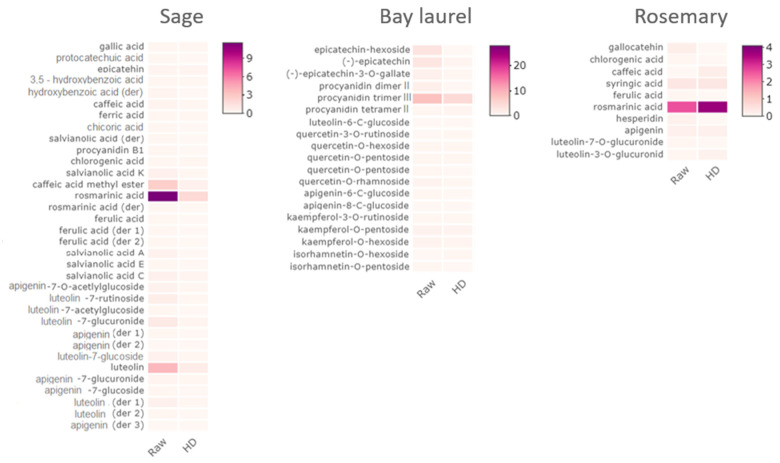
Heatmaps of the phenolic content of sage, bay laurel, and solid rosemary residues, as determined by HPLC. Solid residues remaining after hydrodistillation (HD) were treated with ultrasound and Et-H_2_O (ethanol:water = 1:1 *v*/*v*) as a solvent. Furthermore, hydrodistillation was omitted, and dry plant material was subjected directly to ultrasound-assisted extraction with different solvents (Raw). Similar chemical profiles were obtained after different pre-treatments: HD-RE—reflux extraction, HD-REXPC—reflux extraction assisted with cell wall-degrading enzymes (xylanase, pectinase, and cellulase), HD-US—ultrasound extraction, or with different solvents (Me-H_2_O—methanol:water = 1:1 *v*/*v*; Et-Me-H_2_O—ethanol:methanol:water = 1:1:1 *v*/*v*/*v*), as shown in detail in Supplementary Tables S7–S9. The colors correspond to the mg of the compound per g of the dry plant. Authentic standards used for sage were (+)-catechin, (−)-epicatechin, apigenin, luteolin, rosmarinic acid, caffeic acid, gallic acid, ferulic acid, chicoric acid, chlorogenic acid, procyanidin B1, protocatechinic acid; for bay laurel (+)-catechin, (−)-epicatechin, apigenin, quercetin-3-O-glucoside, quercetin-3-O-rutinoside, kaempferol-3-O-rutinoside, and for rosemary gallic acid, chlorogenic acid, caffeic acid, syringic acid, *p*-coumaric acid, ferulic acid, rosmarinic acid, apigenin, and luteolin.

The chemical composition of sage, rosemary, and bay laurel by-products (Figure 1, Figure 2 and Figure 3, Supplementary Tables S1–S9) obtained after hydrodistillation with different pre-treatments and without pre-treatment were compared by Spearman’s test, i.e., the composition of hydrolates (as analyzed by GC-MS, Supplementary Figure S1), water residues (as analyzed by HPLC, Supplementary Figure S2), and solid residues (as analyzed by HPLC, Supplementary Figure S3). None of the pre-treatments (reflux extraction, HD-RE; reflux extraction assisted with cell wall-degrading enzymes, HD-REXPC; ultrasound extraction, HD-US) significantly affected the chemical composition of hydrodistillation by-products (*p* < 0.05), i.e., the composition was comparable to the no-pretreatment control (HD). Consistent with this, the same pre-treatments did not significantly affect the composition of the essential oils of sage, rosemary, and bay laurel, as reported in our recent article [43]. Other studies have also shown that enzymatic and ultrasonic pre-treatments did not affect the overall composition of essential oils [3,4,6,7,44,45,46], but investigations of the effect of different hydrodistillation pre-treatments on the composition of hydrodistillation by-products are scarce. However, some studies suggest that pre-treatment with different cell wall-degrading enzymes (cellulase, hemicellulase, xylanase, viscozyme) affects the composition of extracts of solid residues of bay laurel [8] and sweet basil [47]. Thus, further studies that compare the effect of different pre-treatments on the composition of hydrodistillation by-products (including hydrolates and water residues) are needed to select the pre-treatment with the most beneficial effect on the final composition of essential oil, as well as the hydrodistillation by-products.

### 2.2. Composition of Sage, Bay Laurel, and Rosemary Hydrolates

Our results have shown that the sage, bay laurel, and rosemary hydrolates are rich in bioactive compounds (Figure 1, Supplementary Tables S1–S3, Supplementary Figure S4), confirming the findings of a few other available studies [20,34,35,48,49].

Sage hydrolates were shown to be rich in oxygenated monoterpenes: among 29 identified components, oxygenated monoterpenes were dominant, with camphor (30.95–34.88%) as the major compound, followed by 1,8-cineole (9.20–17.11%), α-thujone (6.57–16.48%), and borneol (9.60–14.68%), in line with previous reports [20,33,47]. Furthermore, in bay laurel hydrolates, among 17 components identified, the oxygenated monoterpene 1,8-cineole (34.43–46.90 %) was identified as the main compound, followed by camphor (1.36–13.08%) and α–terpineol (6.62–11.55%), similarly to previous data [34,49]. Bay laurel hydrolates also contained significant quantities of phenylpropane derivatives, mainly eugenol, and methyeugenol. Finally, rosemary hydrolates, with 30 components identified by GC-MS, were shown to be rich in oxygenated sesquiterpene berbenone (21.56–42.04%) and also contained a significant abundance of oxygenated monoterpenes, mainly camphor (10.65–17.17%) and 1,8-cineole (8.35–17.08%), also in agreement with the literature [20]. Overall, sage, rosemary, and bay laurel hydrolates consisted mainly of oxygenated compounds, specifically oxygenated monoterpenes in the sage and bay laurel hydrolates and oxygenated sesquiterpenes in the rosemary hydrolates.

Next, the chemical composition of sage, rosemary, and bay laurel hydrolates was compared with the chemical composition of their respective essential oils, reported in our previous study [43] (Figure 1, Tables S1–S3). Hydrolates usually contain <1 g/L of water-soluble volatile organic compounds that originate from the essential oil and remain dissolved in the water phase, but their chemical composition is shifted towards the dominance of more hydrophilic compounds in comparison to the composition of essential oils [2,50]. Consistent with this, our results have shown that oxygenated compounds were dominant in the hydrolates, while monoterpene and sesquiterpene hydrocarbons were dominant in the essential oils. Specifically, hydrolates of sage, bay laurel, and rosemary had a higher proportion of 1,8-cineole (up to 17.11%, 46.90%, and 17.08%, respectively) than their respective essential oils (up to 8.22%, 19.56%, and 9.49%, respectively), as shown in Figure 1 and Supplementary Tables S1–S3. Further, rosemary hydrolates had a higher proportion of oxygenated sesquiterpene berbenone (21.56–42.04%) than rosemary essential oil (up to 21.76%). Sage hydrolates also had a higher proportion of oxygenated monoterpene camphor (30.95–34.88%) than the sage essential oil (up to 17.03%). Further, some minor compounds, such as (E)-hex-2-enal, (E)-hex-2-en-1-ol, hexan-1-ol, oct-1-en-3-ol, octan-3-ol, phenylacetaldehyde, and 2-methoxy-4-vinylphenol in sage, (E)-hex-3-en-1-ol in bay laurel, and (E)-hex-2-enal, (E)-hexen-3-en-1-ol, hexan-1-ol, oct-1-en-3-ol, octan-3-one, benzaldehyde, and phenylacetaldehyde in rosemary, were detected in hydrolates, but not in their respective essential oils. On the other hand, some compounds, mostly sesquiterpenes, that were present in essential oils in significant abundance (like monoterpene α-terpenyl acetate in bay laurel essential oil, sesquiterpenes manool, and viridiflorol in sage essential oil, viridiflorol, trans-caryophyllene, bicyclogermacrene, and β-elemene in bay laurel essential oil) were absent or present in very low amounts, below the detection level, in the hydrolates.

The antibacterial activity of hydrolates of Mediterranean wild plants, including sage and bay laurel, was previously demonstrated [34,49], and this is one of the prerequisites for their possible applications in different fields. Existing studies suggest that one of their possible usages could be in agriculture, for instance, as natural herbicides that could inhibit the seed germination of weeds [51]. Furthermore, hydrolates are already widely used in cosmetics, as they contain valuable bioactive compounds and can be applied topically without dilution [50]. Finally, many of the proposed applications of hydrolates are in the food industry, which is especially promising due to their good organoleptic characteristics and the positive opinion of the public about their use in food products [50,52,53]. For example, hydrolates could be used as convenient sanitizing agents during the preparation of fresh-cut fruits and vegetables, and it has already been demonstrated that washing fresh-cut apple, carrot, and iceberg lettuce samples with hydrolates of sage, bay laurel, and rosemary hydrolates significantly reduced the populations of food spoilage microorganisms on their surface [54,55].

Thus, further examinations of sage, bay laurel, and rosemary hydrolates are needed to develop their possible applications. However, it should be kept in mind that the concentration of bioactive compounds in hydrolates is generally much lower than in the respective essential oils. Thus, their biological activity is expected to be significantly lower than the activity of the respective essential oils [34,50]. In addition, knowing the chemotype of a particular hydrolate is of extreme importance in predicting its biological activity and directing its use toward specific applications. In this study, the dominant components in the hydrolates of wild Mediterranean plants were camphor and 1,8-cineole, known for their antimicrobial properties against many pathogenic and spoilage microbes [56,57].

### 2.3. Composition of Water and Solid Residues of Mediterranean Wild Plants

We used HPLC to analyze the composition of the water residues and the solid residues of sage, bay laurel, and rosemary leaves that remain after hydrodistillation preceded by different pre-treatments (HD-RE, HD-REPCX, HD-US) and without pre-treatment (HD) (Figure 2 and Figure 3, Supplementary Tables S4–S9, Supplementary Figures S5 and S6). 

The water residues of sage, bay laurel, and rosemary leaves are dark, colored solutions with an intense, aromatic odor that accumulates below the steam-distilled biomass due to the partial condensation of the hot steam passing through the biomass. After hydrodistillation, the water residues were collected, and solid residues were subjected to ultrasound-assisted extraction with different solvents: ethanol (ethanol:water = 1:1 *v*/*v*), methanol (methanol:water = 1:1 *v*/*v*), and ethanol–methanol (ethanol:methanol:water = 1:1:1 *v*/*v*/*v*). As a control, hydrodistillation was omitted, and the dry plant material was subjected directly to ultrasound-assisted extraction with different solvents (Raw).

The average total phenolic content in the water residues was 15.1, 20.3, and 39.6 mg/g for rosemary, sage, and bay laurel, respectively. In comparison, the average total phenolic content extracted by ultrasound-assisted extraction with different solvents from the solid residues was three to six times lower (2.6, 4.8, and 6.5 mg/g, respectively) but still in the range of previous reports on the solid residues’ extracts from aromatic plants [47,58,59,60]. The absolute phenolic content of the hydrodistillation by-products and, thus, the concentration of the major biologically active components is one of the key factors determining their biological activity. For example, the antioxidant capacity of rosemary solid residues has been shown to be concentration dependent [24]. Therefore, it may sometimes be necessary to concentrate the residues to obtain higher phenolic concentrations, depending on the desired downstream application.

We show efficient extraction of polyphenols from solid residues by ultrasound in combination with hydroalcoholic solvents (Et-H_2_O—ethanol:water = 1:1 *v*/*v*; Me-H_2_O—methanol:water = 1:1 *v*/*v*; Et-Me-H_2_O—ethanol:methanol:water = 1:1:1 *v*/*v*/*v*). Water/alcohol mixtures have previously been shown to be suitable and environmentally acceptable solvents for extracting polyphenols from sage and other aromatic plants due to the varying polarities of the bioactive constituents [61,62,63]. For instance, when comparing the effects of water, ethanol, and ethanol:water (1:1 *v*/*v*) solvents on isolating phenolic compounds from bay laurel leaves, the best extraction effect was achieved by applying a hydroalcoholic solvent [64]. Further, all tested solvents performed equally well, i.e., the applied solvents did not cause significant variations (*p* > 0.05) in the phenolic composition of the obtained extracts, as confirmed by the Kruskal–Wallis test performed using HPLC data. Also, the extracts of the raw samples (i.e., the dried plant material subjected directly to ultrasound-assisted extraction) had significantly higher total phenolic content (*t*-test, *p* < 0.05) than the extracts of solid hydrodistillation by-products (i.e., solid residues remaining after hydrodistillation subjected to ultrasound-assisted extraction: HD, HD-RE, HD-REXPC, HD-US) (Supplementary Tables S7–S9). However, the difference in total phenolic content of the raw samples and the extracts of solid hydrodistillation by-products was not uniform for different plants, i.e., it was the most pronounced for bay laurel (in average 4× times higher total phenolic content in raw extracts), followed by sage (3×) and finally rosemary (2×). This suggests that the hydrodistillation-assisted extraction of polyphenols was most efficient in bay laurel, thus leaving the smallest amount of polyphenols in the solid residue.

The phenolic composition of the water residues and the solid residue extracts of the same plant was similar, with the same main compounds, only detected in the smaller concentrations in the solid extracts (Figure 2 and Figure 3, Supplementary Tables S4–S9). Thirty-six components were identified in sage water residues, with rosmarinic acid (3.41–5.39 mg/g) as the major compound, followed by caffeic acid methyl ester (1.70–2.61 mg/g dry plant) and epicatechin (1.23–2.06 mg/g dry plant) (Figure 2, Supplementary Table S4). Similarly, 34 components were identified in sage solid residues extracts, again mainly rosmarinic acid (1.16–2.43 mg/g dry plant), followed by luteolin (0.41–0.84 mg/g dry plant) and caffeic acid methyl ester (0.25–0.70 mg/g dry plant) (Figure 3, Supplementary Table S7), which is in agreement with other reports [28,62,64,65].

Bay laurel water and solid residues were dominated by procyanidins. In the water residues, among the 29 identified components, procyanidin dimer I and II (2.06–4.65, 0.79–1.75 mg/g dry plant, respectively), procyanidin tetramer II (2.66–5.43 mg/g dry plant), and procyanidin trimer II, III, and IV (0.89–4.37, 2.57–4.92, 6.03–13.15 mg/g dry plant, respectively) were the most abundant (Figure 2, Supplementary Table S5). Among other components, epicatechin-hexoside (1.51–3.56 mg/g dry plant), (−)-epicatechin (0.86–1.60 mg/g dry plant), and (−)-epicatechin-3-O-gallate (0.35–1.07 mg/g dry plant) were also detected in significant quantities. Similarly, in bay laurel solid residues extracts, among 19 identified components, procyanidin trimer III (1.45–8.67 mg/g) was the major compound, and (−)-epicatechin was also detected in significant quantities (0.20–0.45 mg/g dry plant, Figure 3, Supplementary Table S8). This phenolic composition, dominated by procyanidins and epicatechins, is consistent with the available literature on aqueous, ethanolic, hydroethanolic [63], methanolic [66,67], and acetone [68] extracts of bay laurel leaves.

Finally, the dominant component of the rosemary water and solid residues was rosmarinic acid. In the rosemary water residues, 21 components were identified by HPLC, with rosmarinic acid (2.82–7.57 mg/g), gallocatehin (2.17–4.83 mg/g), *p*-coumaric acid (1.11–2.19 mg/g), and syringic acid (1.40–3.25 mg/g) as the most represented components (Figure 2, Supplementary Table S6). The high observed content of rosmarinic acid is in agreement with an earlier study [27] (up to 8.5 mg rosmarinic acid/g dry leaves in rosemary water residue) and can be explained by good solubility of rosmarinic acid in the water, as a polar protic solvent. Rosmarinic acid was also the most represented in solid residues among 10 components identified by HPLC (Figure 3, Supplementary Table S9) and was detected in the range of concentrations from 0.55 to 3.73 mg/g, in line with previous reports [27,69]. However, in a previous study in which methanol-based Soxhlet extraction was applied to isolate polyphenols from the solid hydrodistillation residue of rosemary leaves, carnosic acid was dominant over rosmarinic acid [27]. Here, carnosic acid was not detected in the solid rosemary residue extract, although its low concentration has been confirmed in the water residue. This is probably mainly due to differences in the extraction solvent (water vs. hydroalcoholic solvent) and different solubility of carnosic acid in those solvents. Carnosic acid was previously found to be more prone to extraction with less polar solvents, such as pure methanol and acetone, due to its low solubility in water [27,70].

Overall, our results demonstrate that water and solid residues obtained in this study are rich in polyphenols: procyanidins in bay laurel and rosmarinic acid in sage and rosemary, the compounds known to have antimicrobial, antioxidant, and other beneficial properties [24,71,72,73]. Furthermore, sage and rosemary water and solid residue are complex mixtures that exhibit antioxidant activity [24,26,74,75,76,77]. Extracts from the solid residue of rosemary also have antibacterial [78] and plant-protective effects (i.e., they repel insects) [24]. To our knowledge, the biological effects of bay laurel solid and water residues have not yet been reported.

Based on such results, solid and water residues of Mediterranean wild plants could find applications in many fields, from the food industry to agriculture. For instance, decoctions of sage and rosemary were already successfully used in marinades for turkey thighs, and since they contained antioxidants, they restrained lipid oxidation and inhibited the development of rancid off-flavors in stored meat [37]. Additionally, they could be used as modifiers for essential oils, as already demonstrated for water residues obtained after essential oil isolation from 15 crops that modified the composition of essential oils of Scotch spearmint (*Mentha* × *gracilis S*. and *Mentha spicata* L.) and peppermint (*Mentha* × *piperita* L.). Their application as a foliar spray during the growth of mints increased essential oil yield and/or the amount of some major compounds [79,80]. In addition, the solid residue extract has the potential to be used as a natural crop protectant, as it can inhibit the feeding of agriculturally important pest insects [24]. Further applications, such as using water residues as natural food preservatives, natural sanitizers for food processing equipment, or as a secondary raw material for the extraction of antioxidants, remain to be tested.

## 3. Conclusions

Essential oils of Mediterranean wild plants are used extensively, but the hydrodistillation residues produced parallel with these essential oils are largely unexplored. We have, for the first time, systematically and simultaneously analyzed all the hydrodistillation by-products of sage, rosemary, and bay laurel, and our results reveal their valuable chemical composition. This research provides a solid foundation for future application studies targeting different industries. We emphasize that the studies preceding the application should confirm the desired biological activities of the hydrodistillation by-products and determine the desired concentrations of phenols and volatiles in the final products.

## 4. Materials and Methods

### 4.1. Extraction Procedures

**Figure 4:**
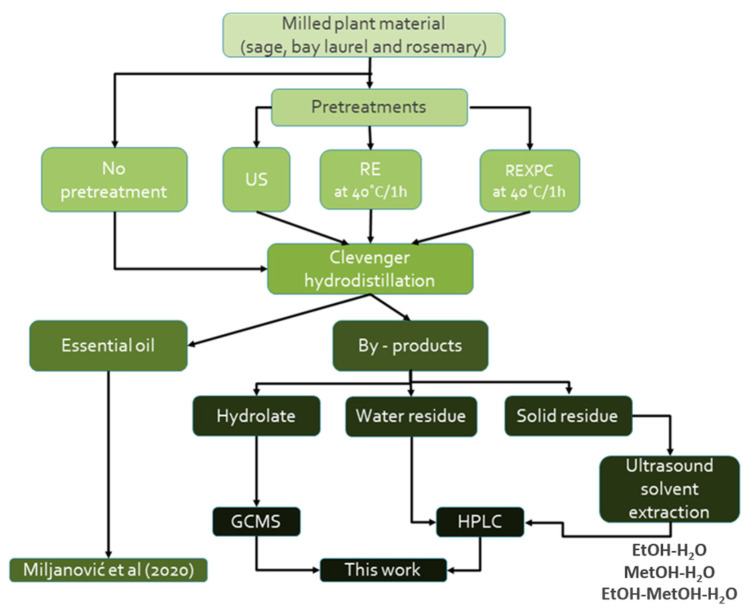
Overview of the extraction protocols used to obtain the hydrodistillation by-products. US—ultrasound extraction; RE—reflux extraction, REXPC—reflux extraction assisted with cell wall-degrading enzymes (xylanase, pectinase, and cellulase).

The collection of plant material (namely, sage leaves, bay laurel, and rosemary) and hydrodistillation (HD) protocols are described in detail in [43] and are schematically represented in Figure 4. We have used the same plant material, of the same age, as in our previous publication [43] and performed three different hydrodistillation procedures per plant, varying in pre-treatments: hydrodistillation with reflux extraction pre-treatment (HD-RE), hydrodistillation with reflux extraction pre-treatment assisted with enzymes (HD-REXPC—combination of xylanase, pectinase, and cellulase) and hydrodistillation with ultrasound pre-treatment (HD-US). HD without pre-treatment served as a negative control. Hydrolate was collected one hour after hydrodistillation was finished. The extraction residue was filtered through the filter paper to separate the water residue (the fluid part) from the solid residue (i.e., the remaining plant material). Solid residues were dried on filter paper at room temperature and then subjected to ultrasound extraction. One gram of solid residue was mixed with 25 mL of solvent: ethanol–water (1:1 *v*/*v*), methanol–water (1:1 *v*/*v*), or ethanol–methanol–water (1:1:1 *v*/*v*/*v*) and treated with 14 mm diameter ultrasonic probe (ultrasonic device UP200Ht, Hielscher, Germany), at 30% of the maximal ultrasonic power during 10 min. The solid residue was then removed by filtering through the filter paper, and the remaining solution was stored. All samples (i.e., hydrolates, water residues, and solid residues) were stored at −18 °C.

### 4.2. Gas Chromatography/Mass Spectrometry (GC-MS) Analysis of the Hydrolate Extracts

The volatile composition was determined by GC-MS. The hydrolate (5 mL) was extracted three times with 1.5 mL of freshly purified diethyl ether (by fractional distillation) by mixing with a magnetic stirrer for 15 min at room temperature for each portion of diethyl ether. The combined diethyl ether extracts were concentrated by fractional distillation up to 2 mL. Extract analyses were carried out by using an Agilent Technologies (Palo Alto, CA, USA) gas chromatograph model 7890 A that was equipped with a mass selective detector (MSD) model 5977E (Agilent Technologies, Palo Alto, CA, USA) and a capillary column (5% phenyl-methylpolysiloxane; 30 m × 0.25 mm, 0.25 μm film thickness). In brief, the injector was 250 °C, and the detector temperature was 300 °C; the column temperature was held at 70 °C for 2 min, and after, was increased to 200 °C at the rate of 3 °C/min and was finally held at 200 °C for 18 min; 1.0 μL of the sample were injected (split ratio 1:50). Helium (1.0 mL/min) was used as a carrier gas. The MSD (EI mode) was at 70 eV, and the scan range was 30–350 amu. Identification of the compounds was based on the comparison of their retention indices (RI), determined relative to the retention times of n-alkanes (C9–C25) (Sigma-Aldrich, St. Louis, MO, USA), with those reported in the literature and their mass spectra with those of authentic compounds or those listed in Wiley 9 and NIST 08 mass spectral libraries. The relative concentrations of components were calculated using the area normalization method without considering the response factors.

### 4.3. High-Performance Liquid Chromatography with Diode Array Detector (HPLC-DAD)

The phenolic composition of the water residues and the extracts of the solid residues was analyzed by high-performance liquid chromatography (HPLC) on an Agilent 1260 system equipped with the Agilent 1260 diode array detector (DAD; Agilent, Santa Clara, CA, USA) with an automatic injector and Chemstation software (version C.01.03). The separation of phenolics was performed using Luna C18 column, particle size 5 µm (250 × 4.6 mm i.d.) (Phenomenex, Aschaffenburg, Germany). The solvent composition and the gradient conditions were previously described [78]. The identification of phenolic compounds was carried out by comparing retention times and characteristic UV/Vis spectra with those of authentic standards, polarity, and the previous literature reports [81,82,83,84]. Phenolic acids and catechins were identified at 280 nm, and flavones and flavonols at 340 nm. Quantitative determination was carried out using the calibration curves of the standards: (+)-catechin (y = 13.241x; R^2^ = 0.9949); protocatechinic acid (y = 27.196x; R^2^ = 0.9989); epicatechin (y = 12.693x, R² = 0.9932); ferulic acid (y = 132.11x; R^2^ = 0.9958); procyanidin B1 (y = 3.57x; R^2^ = 0.9952); chicoric acid: (y = 94.986x; R^2^ = 0.9956); quercetin-3-O-glucoside (y = 37.386x; R^2^ = 1); quercetin-3-O-rutinoside (y = 35.218x; R^2^ = 0.9986); apigenin (y = 107.04x, R² = 0.9969); luteolin (y = 119.39x; R^2^ = 0.9978); rosmarinic acid (y = 31.594x; R^2^ = 0.9965); syringic acid (y = 75.15x; R^2^ = 0.9999); caffeic acid (y = 60.301x; R^2^ = 0.9998); *p*-coumaric acid (y = 135.81x; R^2^ = 1.0000); gallic acid (y = 33.025x; R^2^ = 0.9905), and chlorogenic acid (y = 63.539x; R^2^ = 0.9874). The results were expressed in mg/g of dry matter. For compounds that lacked reference standards, identification was based on mass spectral data and the literature reports of mass fragmentation patterns, while quantification was performed as follows: gallocatechin, 3,5-dihydroxybenzoic acid and hydroxybenzoic acid according to the gallic acid calibration curve; derivatives of ferulic acid according to the ferulic acid calibration curve luteolin-7-O-rutinoside, luteolin-7-acetylglucoside, luteolin-7-glucuronide, luteolin-7-glucoside, luteolin-O-diglucoside according to the luteolin calibration curve; apigenin-7-glucuronide, apigenin-7-glucoside, apigenin-O-pentoside, derivative 1 and 2 of apigenin, apigenin-8-C-glucoside, apigenin-6-C-glucoside, apigenin-7-O-rutinoside, apigenin-7-O-glucuronide, apigenin-acetylglucoside and hesperidin according to the apigenin calibration curve; salvianolic acid K, salvianolic acid I, salvianolic acid A, salvianolic acid E, iso-salvianolic acid C, salvianolic acid C, methyl melitrate A, methyl rosmarinate according to the rosmarinic acid calibration curve; caffeic acid methyl ester according to the caffeic acid calibration curve; epicatechin-hexoside, (−)-epicatechin-3-O-gallate, according to the (−) epicatechin calibration curve; carnosol, carnosic acid according to catechin calibration curve; procyanidin dimer I and II, procyanidin trimer I, II, III, IV and V, procyanidin tetramer I and II according to the procyanidin B1 calibration curve; quercetin-O-hexoside, quercetin-O-pentoside, quercetin-O-rhamnoside, isorhamnetin-O-hexoside, isorhamnetin-O-pentoside, isorhamnetin-O-rhamnoside according to the quercetin-3-O glucoside calibration curve; kaempferol-O-pentoside and kaempferol-O-hexoside according to the kaempherol-3-O rutinoside calibration curve. All analyses have been performed in duplicate, and concentrations of the analyzed compounds are expressed as mg/g (*N* = 2).

### 4.4. Statistical Analyses

All variables were log-transformed (using base 10 logs) to improve the data distribution and homogeneity of variances. Before statistical analysis, all data were checked for normality of distribution using the Shapiro–Wilk test, after which the null hypothesis (that the data are normally distributed) was rejected.

To analyze the possible effects of different pre-treatments on the chemical composition of hydrolates (as determined by GC-MS) and water residues and solid residues (as determined by HPLC) for each plant, we applied Spearman’s nonparametric measure of rank correlation. To test whether the applied solvents significantly affected the phenolic composition of each plant, we performed a Kruskal–Wallis nonparametric test. All analyses were performed using R v. 3.2.0 [85]. The level of significance was set at *p* ≤ 0.05.

To visualize the chemical composition of hydrodistillation by-products, the heatmaps were generated employing the function heatmaply (R package heatmaply v. 0.15.12 https://cran.r-project.org/web/packages/heatmaply/, accessed on 30 May 2021) using the default methods for distance matrix calculation (‘euclidean’).

## Data Availability

The data presented in this study are available in Supplementary Material associated with the manuscript.

## Supplementary Materials

The following supporting information can be downloaded at: https://www.mdpi.com/article/10.3390/plants12132394/s1, Table S1: Chemical composition of sage hydrolates (obtained in this study) compared with the chemical composition of their respective essential oils (reported in [43]). The hydrolates and essential oils were obtained by hydrodistillation with and without different pre-treatments: HD—hy-drodistillation without pre-treatment, HD-RE—hydrodistillation with reflux extraction pre-treatment, HD-US—hydrodistillation with ultrasound extraction pre-treatment, HD-REXPC—hydrodistillation with reflux extraction pre-treatment assisted with cell wall-degrading enzymes (xylanase, pectinase and cellu-lase). RI_E_—experimental retention index on HP-5MS column; RI_L_—retention index from NIST Standard Reference Database 69: NIST Chemistry WebBook (https://webbook.nist.gov/chemistry/, accessed on 30 May 2021). Major compounds (≥5% total peak area in any sample) are marked in blue; Table S2: Chemical composition of bay laurel hydrolates (obtained in this study) compared with the chemical composition of their respective essential oils (reported in [43]). The hydrolates and essential oils were obtained by hydrodistillation with and without different pre-treatments: HD—hy-drodistillation without pre-treatment, HD-RE—hydrodistillation with reflux extraction pre-treatment, HD-US—hydrodistillation with ultrasound extraction pre-treatment, HD-REXPC—hydrodistillation with reflux extraction pre-treatment assisted with cell wall-degrading enzymes (xylanase, pectinase and cellu-lase). RI_E_—experimental retention index on HP-5MS column; RI_L_—retention index from NIST Standard Reference Database 69: NIST Chemistry WebBook (https://webbook.nist.gov/chemistry/, accessed on 30 May 2021). Major compounds (≥5% total peak area in any sample) are marked in blue; Table S3: Chemical composition of rosemary hydrolates (obtained in this study) compared with the chemical composition of their respective essential oils(reported in [43]). The hydrolates and essential oils were obtained by hydrodistillation with and without different pre-treatments: HD—hy-drodistillation without pre-treatment, HD-RE—hydrodistillation with reflux extraction pre-treatment, HD-US—hydrodistillation with ultrasound extraction pre-treatment, HD-REXPC—hydrodistillation with reflux extraction pre-treatment assisted with cell wall-degrading enzymes (xylanase, pectinase and cellu-lase). RI_E_—experimental retention index on HP-5MS column; RI_L_—retention index from NIST Standard Reference Database 69: NIST Chemistry WebBook (https://webbook.nist.gov/chemistry/, accessed on 30 May 2021). Major compounds (≥5% total peak area in any sample) are marked in blue; Table S4: Chemical composition of sage water residues analysed by HPLC. Major compounds (≥3 mg/g in any sample) are marked in blue; Table S5: Chemical composition of bay laurel water residues analysed by HPLC. Major compounds (≥3 mg/g in any sample) are marked in blue; Table S6: Chemical composition of rosemary water residues analysed by HPLC. Major compounds (≥3 mg/g in any sample) are marked in blue; Table S7: Chemical composition of sage solid residue extracts analysed by HPLC. Major compounds (≥3 mg/g in any sample) are marked in blue; Table S8: Chemical composition of bay laurel solid residue extracts analysed by HPLC. Major compounds (≥3 mg/g in any sample) are marked in blue; Table S9: Chemical composition of rosemary solid residue extracts analysed by HPLC. Major compounds (≥3 mg/g in any sample) are marked in blue. Figure S1: Scatter plot showing correlations between different pre-treatments (on the diagonal) regarding the chemical composition of hydrolates. Significant *p*-values based on Spearman’s rank test are shown above the diagonal, while bivariate scatter plots are shown below the diagonal. HD—hydrodistillation without pre-treatment. HD-RE—hydrodistillation pre-treatment with reflux extraction. HD-REXPC—hydrodistillation pre-treatment with reflux extraction assisted with xylanase, pectinase and cellulase. HD-US—hydrodistillation pre-treatment with ultrasound extraction; Figure S2: Scatter plot showing correlations between different pre-treatments (on the diagonal) regarding the chemical composition of water residues. Significant *p*-values based on Spearman’s rank test are shown above the diagonal, while bivariate scatter plots are shown below the diagonal. HD—hydrodistillation without pre-treatment. HD-RE—hydrodistillation pre-treatment with reflux extraction. HD-REXPC—hydrodistillation pre-treatment with reflux extraction assisted with xylanase, pectinase and cellulase. HD-US—hydrodistillation pre-treatment with ultrasound extraction; Figure S3: Scatter plot showing correlations between different pre-treatments (on the diagonal) regarding the chemical composition of solid residue extracts extracted with different solvents. Significant *p*-values based on Spearman’s rank test are shown above the diagonal, while bivariate scatter plots are shown below the diagonal. HD—hydrodistillation without pre-treatment. HD-RE—hydrodistillation pre-treatment with reflux extraction. HD-REXPC—hydrodistillation pre-treatment with reflux extraction assisted with xylanase, pectinase and cellulase. HD-US—hydrodistillation pre-treatment with ultrasound extraction. Figure S4. Total ion chromatograms of sage, bay laurel, and rosemary hydrolates obtained after hydrodistillation without pre-treatment (HD) and determined by GC-MS. Similar chemical profiles were obtained with different pre-treatments: HD-RE—hydrodistillation with reflux extraction pre-treatment, HD-US—hydrodistillation with ultrasound extraction pre-treatment, HD-REXPC—hydrodistillation with reflux extraction pre-treatment assisted with cell wall-degrading enzymes (xylanase, pectinase, and cellulase). Figure S5. HPLC UV-VIS/PDA chromatograms of phenolic compounds were obtained from sage, bay laurel, and rosemary water residues and recorded at 278 nm. Similar chemical profiles were obtained with different pre-treatments: HD-RE—hydrodistillation with reflux extraction pre-treatment, HD-US—hydrodistillation with ultrasound extraction pre-treatment, HD-REXPC—hydrodistillation with reflux extraction pre-treatment assisted with cell wall-degrading enzymes (xylanase, pectinase, and cellulase). Figure S6. HPLC UV-VIS/PDA chromatograms of phenolic compounds were obtained from sage, bay laurel, and solid rosemary residues and recorded at 278 nm. Solid residues remaining after hydrodistillation (HD) were treated with ultrasound and Et-H_2_O (ethanol:water = 1:1 *v*/*v*) as a solvent. Furthermore, hydrodistillation was omitted, and dry plant material was subjected directly to ultrasound-assisted extraction with different solvents (RAW). Similar chemical profiles were obtained after different pre-treatments: HD-RE—reflux extraction, HD-REXPC—reflux extraction assisted with cell wall-degrading enzymes (xylanase, pectinase, and cellulase), HD-US—ultrasound extraction, or with different solvents (Me-H_2_O—methanol:water = 1:1 *v*/*v*; Et-Me-H_2_O—ethanol:methanol:water = 1:1:1 *v*/*v*/*v*).
