# Supplementary material for: Sage, Rosemary, and Bay Laurel Hydrodistillation By-Products as a Source of Bioactive Compounds
Source: Plants (Basel). 2023 Jun 21;12(13):2394. doi: 10.3390/plants12132394 (PMC10346681; doi:10.3390/plants12132394)
Supplement: Supplementary file 1 [file plants-12-02394-s001.zip › plants-2449541-supplementary.pdf]

plants

MDPI

Supplementary Materials of the Manuscript

Sage, Rosemary, and Bay Laurel Hydrodistillation By-Products as a Source of Bioactive Compounds

Anđela Miljanović [1], Maja Dent [1], Dorotea Grbin [1], Sandra Pedisić [1], Zoran Zorić [1], Zvonimir Marijanović [2], Igor Jerković [2] and Ana Bielen [1,*]

Supplementary Tables

Table S1. Chemical composition of sage hydrolates (obtained in this study) compared with the chemical composition of their respective essential oils (reported in Miljanović et al., 2020). The hydrolates and essential oils were obtained by hydrodistillation with and without different pre-treatments: HD – hydrodistillation without pre-treatment, HD-RE – hydrodistillation with reflux extraction pre-treatment, HD-US - hydrodistillation with ultrasound extraction pre-treatment, HD-REXPC – hydrodistillation with reflux extraction pre-treatment assisted with cell wall-degrading enzymes (xylanase, pectinase and cellulase). RI_E – experimental retention index on HP-5MS column; RI_L – retention index from NIST Standard Reference Database *69:* NIST Chemistry WebBook (https://webbook.nist.gov/chemistry/). Major compounds (≥ 5% total peak area in any sample) are marked in **blue**.

Sage		Compound	RI_E	RI_L	% total peak area							
					HD		HD-RE		HD-REXPC		HD-US	
					Hydrolate	Essential oil	Hydrolate	Essential oil	Hydrolate	Essential oil	Hydrolate	Essential oil
Monoterpenes	Monoterpene hydrocarbons	*cis*-salvene	< 900	867	-	0.01	-	0.01	-	0.15	-	0.01
		Tricyclene	929	927	-	0.03	-	0.04	-	0.10	-	0.01
		α-thujene	932	931	-	0.01	-	0.01	-	0.01	-	0.01
		α-pinene	941	941	-	1.25	-	1.03	-	2.64	-	1.83
		Camphene	956	954	-	1.49	-	1.94	-	3.85	-	2.91
		Verbenene	962	967	-	0.04	-	-	-	-	-	-
		Sabinene	979	980	-	0.01	-	0.01	-	0.01	-	0.01

		β-pinene	982	982	-	0.45	-	0.47	-	0.46	-	0.40
		β-myrcene	992	992	-	0.08	-	0.12	-	0.53	-	0.37
		δ-car-3-ene	1014	1013	-	0.05	-	-	-	-	-	-
		α-terpinene	1021	1020	-	0.01	-	0.01	-	0.09	-	0.01
		p-cymene	1030	1030	-	0.19	-	0.22	-	0.99	-	0.90
		Limonene	1034	1032	-	0.10	-	0.32	-	1.46	-	1.16
		γ-terpinene	1063	1062	-	0.03	-	0.01	-	0.08	-	0.01
		α-terpinolene	1091	1085	-	0.06	-	0.01	-	0.05	-	0.01
	Oxygenated monoterpenes	**1,8-cineole**	1038	1038	15.85	5.22	9.20	4.56	17.11	7.23	13.33	8.22
		trans-linalool oxide	1077	1077	0.18	-	0.02	-	0.43	-	0.21	-
		cis-linalool oxide	1091	1091	0.17	-	0.03	-	0.40	-	0.18	-
		Linalool	1104	1104	2.64	2.17	2.22	0.61	0.58	0.37	1.24	0.39
		**α-thujone**	1110	1110	9.74	13.52	6.57	15.33	15.71	18.18	16.48	23.48
		**β-thujone**	1121	1122	3.08	6.57	1.94	6.63	5.51	8.51	5.34	10.22
		Chrysanthenone	1129	1126	0.38	0.21	0.34	-	-	0.04	-	-
		Thujyl alcohol	1141	1146	-	0.45	-	0.38	-	0.23	-	0.27
		**Camphor**	1149	1148	30.99	12.99	30.95	11.69	34.88	12.43	33.54	17.03
		Pinocarvone	1167	1165	0.06	0.07	-	-	-	-	-	-
		**Borneol**	1172	1172	11.89	7.73	14.68	5.77	9.60	4.12	11.44	5.18
		trans-pinocarvone	1179	1173	0.62	0.33	0.62	-	-	-	-	-
		4-terpineol	1182	1184	1.75	0.92	1.99	0.65	1.30	0.44	2.29	0.53
		p-cymen-8-ol	1191	1193	0.96	0.28	1.31	0.18	1.25	0.12	1.15	0.01
		α-terpineol	1195	1190	2.20	0.99	2.82	0.31	0.81	0.18	1.25	0.01
		Myrtenol	1200	1193	-	0.37	-	0.13	-	0.05	-	-
		Homomyrtenol	1208	1212	-	0.39	-	-	-	-	-	-
		trans-carveol	1224	1223	0.25	0.17	0.33	0.11	0.30	0.07	0.32	-
		Geraniol	1261	1267	-	0.11	-	0.01	-	-	0.12	-

		Bornyl acetate	1287	1285	0.11	1.31	-	1.20	0.13	1.09	-	1.00
		trans-sabinyl acetate	1294	1291	-	0.61	-	0.51	-	0.39	-	0.16
		trans-carvyl acetate	1341	1342	-	0.04	-	-	-	-	-	-
		α-terpenyl acetate	1355	1351	-	0.25	-	0.68	-	-	-	-
	**Total monoterpenes**				**80.87**	**58.51**	**73.02**	**52.95**	**88.01**	**63.78**	**86.89**	**74.23**
Sesquiterpenes	Sesquiterpene hydrocarbons	α-ylangene	1373	1372	-	0.06	-	-	-	0.04	-	-
		α-copaene	1378	1377	-	0.18	-	-	-	0.11	-	-
		β-elemene	1393	1387	-	-	-	-	-	-	-	-
		trans-caryophyllene	1421	1420	-	1.21	-	0.73	-	0.51	-	0.30
		α-cadinene	1540	1541	-	0.04	-	0.01	-	0.07	-	0.01
		α-guaiene	1441	1442	-	0.01	-	0.01	-	0.01	-	0.01
		α-humulene	1456	1455	-	3.15	-	3.44	-	3.00	-	1.76
		Alloaromadendrene	1462	1462	-	0.06	-	0.01	-	0.08	-	0.01
		α-amorphene	1478	1482	-	0.32	-	0.19	-	0.25	-	0.01
		β-selinene	1488	1489	-	0.05	-	-	-	-	-	-
		Ledene	1496	1470	-	-	-	-	-	0.16	-	-
		α-muurolene	1501	1500	-	0.13	-	-	-	0.07	-	-
		β-bisabolene	1510	1509	-	0.07	-	-	-	-	-	-
		γ-cadinene	1515	1514	-	0.26	-	0.07	-	0.10	-	0.01
		δ-cadinene	1525	1523	-	0.67	-	0.35	-	0.37	-	0.21
		trans-α-bisabolene	1545	1545	-	-	-	0.01	-	-	-	-
		α-calacorene	1546	1546	-	0.08	-	0.01	-	0.01	-	-
	Oxygenated sesquiterpenes	**Berbenone**	1212	1209	9.34	2.10	14.85	0.01	-	-	-	-
		Spathulenol	1581	1582	-	-		0.10	-	-	-	-
		Caryophyllene oxide	1584	1583	-	1.00	-	0.86	0.20	0.25	0.65	-
		**Viridiflorol**	1594	1593	-	10.13	-	14.39	-	10.64	-	6.52
		α-caryophylladienol	1640	1637	-	0.31	-	-	-	-	-	-

		α-cadinol	1646	1643	-	-	-	0.16	-	0.09	-	-
		β-eudesmol	1654	1648	-	0.10	-	0.18	-	0.06	-	-
		t-muurolol	1658	1659	-	0.19	-	0.27	-	0.09	-	-
		(*E*,*E*)-farnesyl acetone	1919	1918	-	0.15	-	-	-	-	-	-
		Manool	2055	2055	-	8.30	-	13.74	-	11.47	-	14.33
	**Total sesquiterpenes**				**9.34**	**28.57**	**14.85**	**34.54**	**0.20**	**27.38**	**0.65**	**23.17**
Others	Phenylpropane derivatives	Thymol	1296	1294	-	0.04	-	0.36	-	0.06	-	0.01
		Carvacrol	1307	1302	0.09	0.50	0.32	0.01	0.16	0.26	0.15	0.01
		Eugenol	1363	1362	0.14	0.23	0.41	0.36	0.24	2.68	0.92	-
		Methyleugenol	1409	1408	-	0.15	-	0.34	-	-	0.75	-
		Elemicin	1561	1560	-	-		-		-	-	-
	Other compounds	(*E*)-hex-2-enal	< 900	841	0.25	-	0.14	-	0.35	-	0.21	-
		(*E*)-hex-2-en-1-ol	< 900	854	0.03	-	0.01	-	0.05	-	0.03	-
		Hexan-1-ol	< 900	867	0.05	-	0.01	-	0.05	-	0.06	-
		Oct-1-en-3-ol	983	981	0.46		0.34		0.40		0.46	
		Octan-3-ol	998	996	-	-	-	-	-	-	0.04	-
		Phenylacetaldehyde	1049	1049	0.08	-	0.02	-	0.17	-	0.06	-
		2-methoxy-4-vinylphenol	1319	1317	-	-	-	-	0.13	-	-	-
		Methyl jasmonate	1651	1655	-	0.11	-	-	-	-	-	-
		Pentadecanal	1715	1713	-	0.03	-	-	-	-	-	-
		Hexadecan-1-ol	1885	1883	-	0.01	-	-	-	-	-	-
	**Total others**				**1.1**	**1.07**	**1.25**	**1.07**	**1.55**	**0.32**	**2.68**	**0.02**
	**Total:**				**91.31**	**88.15**	**89.12**	**88.56**	**89.76**	**91.48**	**90.22**	**97.42**

Table S2. Chemical composition of bay laurel hydrolates (obtained in this study) compared with the chemical composition of their respective essential oils (reported in Miljanović et al., 2020). The hydrolates and essential oils were obtained by hydrodistillation with and without different pre-treatments: HD – hydrodistillation without pre-treatment, HD-RE – hydrodistillation with reflux extraction pre-treatment, HD-US - hydrodistillation with ultrasound extraction pre-treatment, HD-REXPC – hydrodistillation with reflux extraction pre-treatment assisted with cell wall-degrading enzymes (xylanase, pectinase and cellulase). RI_E – experimental retention index on HP-5MS column; RI_L – retention index from NIST Standard Reference Database *69:* NIST Chemistry WebBook (https://webbook.nist.gov/chemistry/). Major compounds (≥ 5% total peak area in any sample) are marked in **blue**.

Bay laurel		Compound	RI_E	RI_L	% total peak area							
					HD		HD-RE		HD-REPCX		HD-US	
					Hydrolate	Essential oil	Hydrolate	Essential oil	Hydrolate	Essential oil	Hydrolate	Essential oil
Monoterpenes	Monoterpene hydrocarbons	α-thujene	932	931	-[9]	0.04	-	0.04	-	0.01	-	0.09
		α-pinene	941	941	-	0.56	-	1.12	-	1.88	-	1.10
		Camphene	956	954	-	0.28	-	0.21	-	0.02	-	0.23
		Sabinene	979	980	-	1.08	-	1.23	-	2.74	-	1.74
		β-pinene	982	982	-	0.66	-	0.89	-	1.47	-	0.84
		β-myrcene	992	992	-	0.11	-	0.04	-	0.02	-	0.12
		δ-car-3-ene	1014	1013	-	-	-	0.05	-	0.01	-	0.07
		α-terpinene	1021	1020	-	0.05	-	0.01	-	0.02	-	0.15
		p-cymene	1030	1030	-	0.14	-	0.09	-	0.04	-	0.32
		Limonene	1034	1032	-	0.01	-	0.01	-	0.62	-	0.76
		γ-terpinene	1063	1062	-	0.08	-	0.07	-	0.02	-	0.28
		α-terpinolene	1091	1085	-	0.03	-	-	-	-	-	0.08
	Oxygenated monoterpenes	**1,8-cineole**	1038	1038	46.90	13.26	36.39	16.86	34.43	27.10	40.53	19.56
		cis-sabinene hydrate	1073	1074	-	0.10	-	0.33	-	-	-	0.22
		trans-linalool oxide	1077	1077	-	-	-	-	0.13		-	-
		cis-linalool oxide	1091	1091	-	-	-	-	0.10	-	-	-
		**Linalool**	1104	1104	8.75	7.80	4.47	6.39	7.66	4.91	4.42	4.92
		α-thujone	1110	1110	1.60	3.51	-	1.09	0.10	0.09	-	1.18
		β-thujone	1121	1122	0.61	1.97	-	0.45	-	0.06	0.31	0.53
		cis-*p*-menth-2-en-1-ol	1127	1125	-	-	-	-	-	-	-	0.06

		Chrysanthenone	1129	1126	-	-	-	0.13	-	-	-	0.06
		Thujyl alcohol	1141	1146	-	0.09	-	-	-	-	-	-
		Terpenene-1-ol	1146	1144	-	0.11	-	-	-	-	-	0.07
		**Camphor**	1149	1148	4.23	2.90	13.08	2.69	1.36	1.57	5.56	1.93
		Pinocarvone	1167	1165	-	-	-	0.09	-	-	-	0.12
		**Borneol**	1172	1172	3.27	2.04	7.99	3.05	3.19	1.74	4.38	1.55
		Isopinocamphone	1178	1181	-	-	-	0.20	-	-	-	-
		4-terpineol	1182	1184	4.66	1.83	3.87	1.93	5.09	2.65	4.39	1.71
		p-cymen-8-ol	1191	1193	-	-	-	0.08	0.16	-	0.33	0.05
		**α-terpineol**	1195	1190	11.1	3.76	6.62	4.25	11.55	5.26	9.67	3.84
		Myrtenol	1200	1193	-	-	-	0.14	-	-	-	-
		Homomyrtenol	1208	1212	-	-	-	0.21	-	-	-	-
		Nerol	1233	1232	-	0.35	-	0.48	0.37	0.56	-	0.21
		Linalyl acetate	1259	1259	-	0.29	-	0.07	-	0.01	-	0.02
		Geraniol	1261	1267	-	-	-	0.10	-	-	-	-
		Bornyl acetate	1287	1285	-	1.97	-	0.83	-	0.83	-	0.64
		trans-sabinyl acetate	1294	1291	-	0.15	-	-	-	-	-	
		δ-terpinyl acetate	1319	1316	-	0.90	-	0.63	-	0.46	-	0.54
		2-acetoxy-1,8-cineole	1344	1344	-	0.16	-	0.19	-	0.02	-	0.12
		**α-terpenyl acetate**	1355	1351	-	16.94	-	15.71	-	18.16	-	15.84
		Neryl acetate	1367	1367	-	0.23	-	0.13	-	0.01	-	0.12
		trans-cinnamyl acetate	1449	1448	-	0.20	-	-	-	-	-	-
		Spathulenol	1581	1576	-	2.40	-	1.52	-	1.18	-	1.62
		Manool	2055	2055	-	2.10	-	1.03	-	-	-	-
	**Total monoterpenes**				**81.12**	**66.1**	**72.42**	**62.34**	**64.14**	**70.79**	**69.59**	**60.69**
Sesquiterpenes	Sesquiterpenes hydrocarbons	α-ylangene	1373	1372	-	0.15	-	0.14	-	0.01	-	0.26
		α-copaene	1378	1377	-	0.06	-	0.10	-	0.01	-	0.09

		β-cubebene	1391	1391	-	0.06	-	-	-	-	-	0.04
		β-elemene	1393	1387	-	0.50	-	0.40	-	0.50	-	1.53
		trans-caryophyllene	1421	1420	-	1.40	-	2.47	-	3.99	-	2.92
		α-guaiene	1441	1442	-	0.30	-	0.31	-	0.49	-	0.46
		Guaia-3,7-diene	1446	1440	-	0.07	-	0.12	-	0.01	-	0.18
		α-humulene	1456	1455	-	0.67	-	0.74	-	0.46	-	0.66
		Alloaromadendrene	1462	1462	-	0.13	-	0.14	-	0.02	-	0.18
		α-amorphene	1478	1482	-	0.10	-	0.14	-	0.01	-	0.12
		Germacrene D	1482	1480	-	0.30	-	0.37	-	0.02	-	0.63
		β-selinene	1488	1489	-	0.38	-	0.33	-	0.51	-	0.59
		Bicyclogermacrene	1496	1496	-	1.18	-	0.83	-	1.19	-	1.40
		Germacrene A	1505	1504	-	0.71	-	0.58	-	1.25	-	0.87
		γ-cadinene	1515	1514	-	0.25	-	0.33	-	0.02	-	0.52
		δ-cadinene	1525	1523	-	0.44	-	0.66	-	0.58	-	0.81
		trans-α-bisabolene	1545	1545	-	0.32	-	0.25	-	0.02	-	0.20
	Oxygenated sesquiterpenes	**Berbenone**	1212	1209	-	-	10.63	1.49	1.11	1.17	10.30	0.70
		Elemol	1553	1551	-	0.13	-	0.09	-	0.01	-	0.15
		Nerolidol	1568	1565	-	0.19	-	0.06	-	0.02	-	0.01
		Caryophyllene oxide	1584	1583	-	0.70	-	1.39	-	1.19	-	1.07
		Globulol	1586	1590	-	0.18	-	-	-	-	-	-
		Viridiflorol	1594	1593	-	4.14	-	1.64	-	0.02	-	1.16
		Isospathulenol	1642	1640	-	0.41	-	-	-	-	-	0.24
		α-cadinol	1646	1643	-	0.35	-	0.37	-	0.01	-	0.39
		β-eudesmol	1654	1648	-	1.11	-	0.67	-	0.02	-	0.78
		α-eudesmol	1657	1652	-	1.25	-	-	-	-	-	-
		t-muurolol	1658	1659	-	-	-	0.83	-	0.01	-	1.06
	**Total sesquiterpenes**				**0.00**	**15.63**	**10.63**	**14.45**	**1.11**	**11.54**	**10.30**	**17.02**

Others	Phenylpropane derivatives	p-allylanisole	1199	1197	-	0.08	-	-	-	-	-	0.08
		3-phenylprop-2-enal	1275	1278	-	0.06	-	-	-	-	-	-
		Thymol	1296	1294	-	0.05	-	-	-	-	-	-
		Carvacrol	1307	1302	-	0.16	-	-	-	-	-	-
		**Eugenol**	1363	1362	8.11	2.76	8.04	6.33	19.97	8.20	13.99	5.64
		**Methyleugenol**	1409	1408	4.40	7.15	2.09	8.52	8.19	9.33	3.30	7.95
		cis-methylisoeugenol	1500	1494	-	0.54	-	-	-	-	-	0.32
		Elemicin	1561	1560	-	0.32	-	0.48	-	0.01	-	0.37
	Other compounds	(Z)-hex-3-en-1-ol	< 900	855	-	-	1.01	-	1.52	-	1.24	-
		Nonan-2-one	1094	1091	-	-	-	-	-	-	-	-
		Nonanal	1105	1102	0.63	-	-	-	-	-	-	-
		Decanal	1207	1204	-	-	-	-	-	-	-	-
		Undecan-2-one	1295	1291	-	-	-	0.17	-	-	-	0.12
		Pentadecan-2-one	1699	1699	-	-	-	-	-	-	-	0.09
		Hexadecanal	1818	1820	-	-	-	-	-	-	-	-
	**Total others**				**13.14**	**11.12**	**11.14**	**15.5**	**29.68**	**17.54**	**18.53**	**14.57**
	**Total:**				**94.26**	**92.85**	**94.19**	**92.29**	**94.93**	**99.87**	**98.65**	**92.28**

Table S3. Chemical composition of rosemary hydrolates (obtained in this study) compared with the chemical composition of their respective essential oils (reported in Miljanović et al. 2020). The hydrolates and essential oils were obtained by hydrodistillation with and without different pre-treatments: HD – hydrodistillation without pre-treatment, HD-RE – hydrodistillation with reflux extraction pre-treatment, HD-US - hydrodistillation with ultrasound extraction pre-treatment, HD-REXPC – hydrodistillation with reflux extraction pre-treatment assisted with cell wall-degrading enzymes (xylanase, pectinase and cellulase). RI – experimental retention index on HP-5MS column; RI_L – retention index from NIST Standard Reference Database *69:* NIST Chemistry WebBook (https://webbook.nist.gov/chemistry/). Major compounds (≥ 5% total peak area in any sample) are marked in **blue**.

**Rosemary**		**Compound**	**RI_E**	**RI_L**	**% total peak area**							
					**HD**		**HD-RE**		**HD-REPCX**		**HD-US**	
					**Hydrolate**	**Essential oil**	**Hydrolate**	**Essential oil**	**Hydrolate**	**Essential oil**	**Hydrolate**	**Essential oil**
Monoterpenes	Monoterpene hydrocarbons	**α-pinene**	941	941	0.01	2.25	-	3.28	-	3.36	-	5.88
		Camphene	956	954	-	0.47	-	0.62	-	0.59	-	1.19
		Verbenene	962	967	-	-	-	-	-	0.19	-	0.35
		Sabinene	979	980	-	-	-	-	-	0.08	-	-
		β-pinene	982	982	-	-	-	0.27	-	0.28	-	0.29
		β-myrcene	992	992	-	-	-	-	-	-	-	-
		p-cymene	1030	1030	-	0.01	-	0.42	-	0.15	-	1.03
		δ-car-3-ene	1014	1013	-	-	-	0.33	-	0.21	-	0.51
		Limonene	1034	1032	-	0.01	-	0.58	-	0.25	-	1.49
	Oxygenated monoterpenes	**1,8-cineole**	1038	1038	8.35	4.34	10.36	9.49	15.98	7.44	17.08	4.24
		**Linalool**	1104	1104	3.64	5.00	3.57	5.75	5.86	5.57	3.43	1.38
		Filifolone	1105	1103	0.21	-	0.17	0.38	0.13	0.29	0.10	0.29
		α-thujone	1110	1110	0.11	-	0.07	0.72	0.12	0.61	0.85	3.47
		β-thujone	1121	1122	0.02	-	0.01	0.38	0.02	0.35	0.28	1.49
		Chrysanthenone	1129	1126	0.54	0.20	0.56	0.72	0.34	0.58	0.29	0.14
		**Camphor**	1149	1148	16.65	15.38	17.17	13.82	10.65	11.56	11.50	9.01
		Pinocarvone	1167	1165	0.017	-	0.02	0.73	0.07	0.20	0.01	0.58
		**Borneol**	1172	1172	10.72	24.38	10.77	12.03	7.75	11.26	6.85	5.39
		Isopinocamphone	1178	1181	0.79	0.69	0.20	1.52	0.42	1.15	0.32	0.64
		4-terpineol	1182	1184	1.50	1.00	1.49	1.70	2.91	1.24	2.34	0.52

		p-cymen-8-ol	1191	1193	-	-	-	-	-	0.24	-	-
		**α-terpineol**	1195	1190	-	5.04	-	4.17	-	3.34	-	0.83
		Myrtenol	1200	1193	-	1.28	-	0.56	-	0.53	-	-
		Homomyrtenol	1208	1212	-	2.39	-	-	-	1.05	-	0.23
		trans-carveol	1225	1223	0.21	-	0.21	-	0.12	-	0.10	-
		Nerol	1233	1232	0.15	-	0.12	-	0.29	0.27	0.12	-
		Geraniol	1261	1267	-	-	-	-	0.14	0.21	-	-
		Bornyl acetate	1287	1285	-	-	-	0.41	-	0.23	-	0.47
		α-terpenyl acetate	1355	1351	-	-	-	3.31	-	2.38	-	-
		Spathulenol	1581	1576	-	-	-	0.64	-	1.28	-	-
	**Total monoterpenes**				**42.90**	**62.44**	**44.72**	**61.83**	**44.80**	**52.17**	**43.27**	**39.42**
Sesquiterpenes	Sesquiterpene hydrocarbons	β-elemene	1393	1387	-	-	-	-	-	0.22	-	-
		α-copaene	1378	1377	-	-	-	-	-	0.13	-	-
		trans-caryophyllene	1421	1420	-	1.53	-	2.84	-	1.47	-	0.56
		α-guaiene	1441	1442	-	-	-	-	-	0.11	-	-
		α-humulene	1456	1455	-	-	-	0.66	-	0.45	-	1.24
		Alloaromadendrene	1462	1462	-	-	-	-	-	-	-	-
		α-amorphene	1478	1482	-	-	-	-	-	0.13	-	-
		β-selinene	1488	1489	-	-	-	-	-	0.13	-	-
		α-muurolene	1501	1499	-	-	-	-	-	0.14	-	-
		γ-cadinene	1515	1514	-	0.01	-	0.43	-	0.28	-	0.02
		α-calacorene	1546	1546	-	-	-	-	-	0.19	-	-
		Germacrene A	1505	1504	-	-	-	0.40	-	-	-	-
		δ-cadinene	1525	1523	-	0.81	-	1.22	-	0.60	-	0.69
	Oxygenated sesquiterpenes	**Berbenone**	1212	1209	42.04	21.76	40.05	12.17	21.56	9.93	38.86	5.56
		Spathulenol	1581	1582	-	-	-	0.64	0.17	1.28	-	-
		Caryophyllene oxide	1584	1583	-	-	-	0.67	-	0.92	-	-

		Viridiflorol	1594	1593	-	-	-	-	-	1.85	-	14.32
		α-caryophylladienol	1640	1637	-	-	-	-	-	0.83	-	1.11
		α-cadinol	1646	1643	-	-	-	0.63	-	0.82	-	-
		β-eudesmol	1654	1648	-	-	-	0.84	-	1.15	-	-
		t-muurolol	1658	1659	-	-	-	1.54	-	1.84	-	0.13
		Manool	2055	2055	-	-	-	0.45	-	1.71	-	22.05
	**Total sesquiterpenes**				**42.04**	**24.11**	**40.05**	**23.53**	**21.73**	**25.1**	**38.86**	**45.68**
Others	Phenylpropane derivatives	Thymol	1296	1294	-	-	-	-	-	0.20	-	-
		Carvacrol	1307	1302	0.17	-	0.10	-	0.17	0.32	0.12	-
		**Eugenol**	1363	1362	0.36	-	0.10	5.70	11.49	4.24	4.90	-
		**Methyleugenol**	1409	1408	0.25	0.01	0.09	3.56	6.36	2.82	2.71	-
		Elemicin	1561	1560	-	-	-	0.46	0.49	0.39	-	-
	Other compounds	(E)-hex-2-enal	< 900	841	0.04	-	-	-	-	-	-	-
		(Z)-hex-3-en-1-ol	< 900	854	0.01	-	0.04	-	0.21	-	0.12	-
		Hexan-1-ol	< 900	867	0.02	-	0.02	-	-	-	-	-
		Benzaldehyde	968	970	0.02	-	0.03	-	0.01	-	0.01	-
		Oct-1-en-3-ol	983	981	0.32	-	0.35	-	0.16	-	0.19	-
		Octan-3-one	988	985	0.08	-	0.08	-	0.01	-	0.01	-
		Phenylacetaldehyde	1050	1049	0.02	-	0.04	-	0.14	-	0.01	-
		Methyl jasmonate	1651	1655	-	-	-	-	-	0.62	-	-
		Hexadecan-1-ol	1885	1883	-	-	-	-	-	0.13	-	-
	**Total others**				**1.29**	**0.01**	**0.85**	**9.72**	**19.04**	**8.72**	**8.07**	**0**
	**Total:**				**86.23**	**86.56**	**85.62**	**95.07**	**85.57**	**85.99**	**90.02**	**85.1**

Table S4. Chemical composition of sage water residues analysed by HPLC. Major compounds (≥ 3 mg/g in any sample) are marked in **blue**.

Sage	ω (mg/g)			
	**HD**	**HD-RE**	**HD-REPCX**	**HD-US**
Catechin*	0.88	1.18	1.26	0.70
Gallic acid*	0.53	0.36	0.30	0.30
Protocatechinic acid*	N.D.	N.D.	0.01	N.D.
Chicepicatechin*	1.84	1.23	2.06	1.49
3,5 - dihydroxybenzoic acid	0.05	0.04	0.06	N.D.
Hydroxybenzoic acid	0.62	0.49	0.89	0.66
Caffeic acid*	0.09	0.08	0.14	0.09
Procyanidin B1*	1.33	0.68	1.27	1.25
Chicoric acid*	0.02	0.02	0.03	0.02
Chlorogenic acid*	0.49	0.49	1.08	0.43
Salvianolic acid K	0.84	0.66	1.46	0.87
Caffeic acid methyl ester	2.26	1.70	2.61	2.46
**Rosmarinic acid***	4.63	3.41	5.01	5.39
Ferulic acid*	0.12	0.04	0.08	0.12
Salvianolic acid I	0.04	0.03	0.11	0.05
Methyl melitrate A	0.04	0.02	0.06	0.04
Ferulic acid (der 1)	0.01	0.01	0.02	0.01
Ferulic acid (der 2)	0.08	0.06	0.10	0.09
Ferulic acid (der 3)	0.01	0.01	0.01	0.01
Salvianolic acid A	0.49	0.37	0.57	0.53
Salvianolic acid E	0.04	0.03	0.02	0.05
Iso - salvianolic acid C	0.14	0.10	0.20	N.D.
Salvianolic acid C	0.01	N.D.	0.10	N.D.
Apigenin-7-O-acetlylglucoside	0.19	0.14	0.26	0.20
Luteolin-7-rutinoside	0.52	0.38	0.72	0.56

Luteolin-7-acetylglucoside	0.86	0.60	1.13	0.89
Luteolin-7-glucuronide	0.03	0.03	0.05	0.01
Luteolin-7-glcucoside	0.33	0.23	0.44	0.37
Luteolin*	1.79	1.30	2.20	1.93
Apigenin-7-glucuronide	1.61	1.22	2.00	1.75
Apigenin-7-glucoside	0.12	0.10	0.18	0.12
Apigenin-O-pentoside	0.17	0.12	0.22	0.18
Apigenin (der 1)	0.13	0.09	0.18	0.13
Apigenin (der 2)	0.01	0.01	0.03	0.03
**TOTAL**	**20.31**	**15.21**	**24.82**	**20.69**

HD - without pre-treatment. HD-RE - pre-treatment with reflux extraction, HD-REXPC - pre-treatment with reflux extraction assisted with xylanase, pectinase and cellulase, HD-US – pre-treatment with ultrasound extraction. N.D. - not detected. *Authentic standards used: (+)-catechin, (-)-epicatechin, apigenin, luteolin, rosmarinic acid, caffeic acid, gallic acid, ferulic acid, chicoric acid, chlorogenic acid, procyanidin B1, protocatechinic acid.

Table S5. Chemical composition of bay laurel water residues analysed by HPLC. Major compounds (≥ 3 mg/g in any sample) are marked in **blue**.

**Bay laurel**	**W (mg/g)**			
	**HD**	**HD-RE**	**HD-REPCX**	**HD-US**
**Epicatechin-hexoside**	1.51	2.86	3.56	2.82
(-)-epicatechin*	0.86	1.47	1.57	1.60
(-)-epicatechin-3-*O*-gallate	0.35	0.64	1.07	0.97
(+)-catechin*	0.67	1.24	2.08	1.62
**Procyanidin dimer I**	2.06	3.15	4.65	3.80
Procyanidin dimer II	0.98	0.79	1.75	1.34
Procyanidin trimer I	0.68	2.12	1.34	2.39
Procyanidin trimer II	0.89	3.42	1.45	4.37
**Procyanidin trimer III**	2.57	4.19	4.92	3.85
**Procyanidin trimer IV**	6.03	10.49	13.15	11.63

Procyanidin trimer V	0.16	N.D.	0.21	0.36
Procyanidin tetramer I	0.42	0.81	1.19	0.83
**Procyanidin tetramer II**	2.66	4.98	4.93	5.43
Apigenin-8-*C*-glucoside	0.04	0.05	0.07	0.06
Apigenin-6-*C*-glucoside	0.14	0.16	0.17	0.37
Quercetin-3-*O*-glucoside*	0.38	0.42	0.37	0.65
Quercetin-3-*O*-rutinoside*	0.30	0.41	0.54	N.D.
Quercetin-*O*-hexoside	0.32	0.49	0.69	0.72
Quercetin-*O*-pentoside	0.54	0.92	0.93	0.28
Quercetin-*O*-rhamnoside	0.60	0.87	0.95	0.89
Kaempferol-*O*-pentoside	0.08	0.13	0.23	1.09
Kaempferol-*O*-hexoside	0.28	0.35	0.64	0.51
Kaempferol-3-*O*-rutinoside*	0.16	0.25	0.27	0.26
Isorhamnetin-*O*-hexoside	0.08	0.09	0.11	0.32
Isorhamnetin-*O*-pentoside	0.02	0.02	0.01	0.03
Isorhamnetin-*O*-rhamnoside	0.31	0.39	0.71	0.56
**TOTAL**	**23.09**	**40.71**	**47.56**	**46.75**

HD - without pre-treatment. HD-RE - pre-treatment with reflux extraction, HD-REXPC - pre-treatment with reflux extraction assisted with xylanase, pectinase and cellulase. HD-US – pre-treatment with ultrasound extraction. N.D. - not detected. *Authentic standards used: (+)-catechin, (-)-epicatechin, apigenin, quercetin-3-O-glucoside, quercetin-3-O-rutinoside, kaempferol-3-*O*-rutinoside

Table S6. Chemical composition of rosemary water residues analysed by HPLC. Major compounds (≥ 3 mg/g in any sample) are marked in **blue**.

**Rosemary**	**W (mg/g)**			
	**HD**	**HD-RE**	**HD-REPCX**	**HD-US**
Gallic acid*	0.27	N.D.	0.24	N.D.
Chlorogenic acid*	0.30	0.08	0.14	N.D.
Caffeic acid*	0.84	1.20	0.81	0.44

**Syringic acid***	3.25	2.04	2.31	1.40
p-coumaric acid*	2.19	1.11	1.69	1.32
Carnosol	0.10	N.D.	N.D.	N.D.
Carnosic acid	0.34	N.D.	0.16	0.11
Ferulic acid*	0.01	N.D.	N.D.	N.D.
**Rosmarinic acid***	7.57	2.82	5.16	3.64
Methyl rosmarinate	0.41	0.10	0.53	0.50
Hesperidin	0.15	N.D.	N.D.	N.D.
**Gallocatechin**	4.83	2.95	3.79	2.17
Luteolin-3-*O*-glucuronide	0.21	0.05	0.14	0.12
Luteolin-7-O-rutinoside	0.11	0.06	0.09	0.07
Luteolin-*O*-diglucoside	0.03	0.03	0.02	N.D.
Apigenin*	0.20	0.50	0.16	0.16
Apigenin-7-*O*-rutinoside	0.70	N.D.	0.82	0.64
Apigenin-*O*-pentoside	0.12	0.03	N.D.	N.D.
Apigenin-7-*O*-glucuronide	0.15	0.06	0.06	0.04
Apigenin-7-glucoside	0.20	0.03	0.16	0.16
Apigenin-acetylglucoside	0.14	0.03	0.08	0.06
**TOTAL**	**22.12**	**11.09**	**16.36**	**10.83**

HD - without pre-treatment. HD-RE - pre-treatment with reflux extraction. HD-REXPC - pre-treatment with reflux extraction assisted with xylanase, pectinase and cellulase. HD-US – pre-treatment with ultrasound extraction. N.D. - not detected. *Authentic standards used: gallic acid, chlorogenic acid, caffeic acid, syringic acid, *p*--coumaric acid, ferulic acid, rosmarinic acid, apigenin, luteolin.

Table S7. Chemical composition of sage solid residue extracts analysed by HPLC. Major compounds (≥ 3 mg/g in any sample) are marked in **blue**.

Sage	w (mg/g)														
	Et-H_2O					Me-H_2O					Et-Me-H_2O				
	RAW	HD	HD-RE	HD-REXPC	HD-US	RAW	HD	HD-RE	HD-REXPC	HD-US	RAW	HD	HD-RE	HD-REXPC	HD-US
Gallic acid*	0.07	0.13	0.19	0.24	0.19	0.13	0.16	0.18	0.18	0.23	0.14	0.06	0.21	0.22	0.07
Protocatechuic acid	0.05	0.02	N.D.	N.D.	N.D.	N.D.	N.D.	N.D.	N.D.	N.D.	0.06	N.D.	N.D.	N.D.	N.D.
Epicatechin*	0.18	0.18	0.19	0.23	0.19	N.D.	0.19	0.21	0.13	0.17	N.D.	0.24	0.18	0.26	0.22
3.5 - hydroxybenzoic acid (der)	0.15	0.05	0.09	0.12	0.14	0.13	0.07	0.11	0.14	0.11	0.26	0.05	0.08	0.10	0.07
Hydroxybenzoic acid (der)	0.21	N.D.	N.D.	N.D.	0.04	N.D.	N.D.	N.D.	N.D.	0.04	0.11	N.D.	0.05	0.07	0.05
Caffeic acid*	0.27	0.04	0.03	0.05	0.04	0.06	0.03	0.04	0.04	0.04	0.06	0.03	0.03	0.05	0.04
Chicoric acid	0.04	0.03	0.03	0.04	0.03	0.19	0.03	0.03	0.03	0.03	0.19	0.03	0.03	0.04	0.03
Salvianolic acid (der)	0.09	N.D.	N.D.	N.D.	N.D.	N.D.	0.13	N.D.	N.D.	N.D.	N.D.	N.D.	N.D.	N.D.	N.D.
Procyanidin B1*	0.17	N.D.	N.D.	N.D.	N.D.	N.D.	N.D.	N.D.	N.D.	N.D.	N.D.	N.D.	N.D.	N.D.	N.D.
Chlorogenic acid*	0.07	0.12	0.20	0.28	0.19	0.37	0.07	0.19	0.13	0.13	0.41	0.17	0.27	0.37	0.22
Salvianolic acid K	0.49	0.09	0.13	0.17	0.16	0.06	0.12	0.12	0.19	0.14	0.45	0.19	0.19	0.20	0.17
Caffeic acid methyl ester	2.69	0.50	0.52	0.52	0.70	0.31	0.25	0.47	0.32	0.53	0.50	0.61	0.45	0.49	0.57
**Rosmarinic acid***	11.53	2.00	1.43	1.83	1.84	2.56	1.71	1.36	1.16	1.41	4.30	2.43	1.78	1.68	2.32
Rosmarinic acid (der)	0.10	N.D.	N.D.	N.D.	N.D.	N.D.	N.D.	N.D.	N.D.	0.02	N.D.	N.D.	N.D.	N.D.	N.D.
Ferulic acid*	0.09	0.01	0.02	0.02	N.D.	0.02	0.01	0.01	0.01	0.01	0.02	0.02	0.02	0.01	0.01
Ferulic acid (der 1)	0.04	0.03	0.02	0.02	N.D.	0.03	0.02	0.02	0.02	0.03	0.05	0.02	0.02	0.02	0.02
Ferulic acid (der 2)	0.16	0.02	0.02	0.03	N.D.	0.01	0.02	0.02	0.02	0.02	0.02	0.03	0.03	0.03	0.03
Salvianolic acid A	0.32	N.D.	N.D.	N.D.	N.D.	N.D.	N.D.	N.D.	N.D.	N.D.	N.D.	N.D.	N.D.	N.D.	N.D.
Salvianolic acid E	0.08	N.D.	N.D.	N.D.	N.D.	N.D.	N.D.	N.D.	N.D.	N.D.	N.D.	N.D.	N.D.	N.D.	N.D.
Salvianolic acid C	0.43	0.18	0.02	0.17	0.11	0.38	0.19	0.17	0.08	0.09	0.52	0.30	0.22	0.20	0.11
Apigenin-7-O-acetlylglucoside	0.35	0.04	0.03	0.05	0.04	0.24	0.03	0.03	0.04	0.04	0.26	0.04	0.03	0.05	0.04
Luteolin-7-rutinoside	0.64	0.08	0.10	0.14	0.12	0.38	0.07	0.09	0.10	0.10	0.33	0.09	0.10	0.11	0.10
Luteolin-7-acetylglucoside	0.10	0.03	0.04	0.06	0.05	0.08	0.03	0.03	0.05	0.04	0.08	0.04	0.04	0.06	0.04

Luteolin-7-glucuronide	1.01	0.19	0.27	0.27	0.22	0.20	0.12	0.22	0.03	0.04	0.31	0.34	0.29	0.30	0.30
Apigenin (der 1)	0.15	0.01	0.01	0.01	0.01	0.09	0.01	0.01	0.01	0.01	0.06	0.01	0.01	0.01	0.01
Apigenin (der 2)	0.03	N.D.	0.02	0.04	0.04	0.03	0.02	N.D.	N.D.	N.D.	N.D.	N.D.	N.D.	N.D.	N.D.
Luteolin-7-glucoside	0.34	0.07	0.07	0.11	0.10	0.20	0.06	0.07	0.07	0.08	0.23	0.10	0.08	0.11	0.10
**Luteolin***	3.80	0.78	0.61	0.76	0.68	1.77	0.59	0.53	0.41	0.46	2.00	0.84	0.67	0.80	0.77
Apigenin-7-glucuronide	0.20	0.04	0.04	0.04	0.03	0.14	0.03	0.03	0.03	0.03	0.15	0.05	0.04	0.04	0.03
Apigenin-7-glucoside	0.07	0.02	0.03	0.03	0.03	0.03	0.02	0.02	0.02	0.02	0.04	0.03	0.03	0.04	0.04
Luteolin (der 1)	0.44	0.11	0.15	0.19	0.18	0.38	0.08	0.15	0.11	0.14	0.17	0.16	0.14	0.19	0.20
Luteolin (der 2)	0.04	0.02	0.07	0.05	0.02	0.06	N.D.	0.01	0.01	N.D.	0.20	0.04	0.11	0.09	0.03
Apigenin (der 3)	0.02	0.02	0.01	N.D.	N.D.	0.03	N.D.	N.D.	N.D.	N.D.	0.03	0.01	0.01	0.01	0.01
**TOTAL**	**24.47**	**4.85**	**4.35**	**5.51**	**5.15**	**7.87**	**4.06**	**4.12**	**3.34**	**3.97**	**10.98**	**5.93**	**5.13**	**5.57**	**5.65**

RAW - dry plant material subjected directly to the ultrasound assisted extraction, without hydrodistillation. HD - hydrodistillation without pre-treatment. HD-RE - hydrodistillation pre-treatment with reflux extraction. HD-REXPC - hydrodistillation pre-treatment with reflux extraction assisted with xylanase, pectinase and cellulase. HD-US – hydrodistillation pre-treatment with ultrasound extraction. N.D. - not detected. *Authentic standards used: (+)-catechin, (-)-epicatechin, apigenin, luteolin, rosmarinic acid, caffeic acid, gallic acid, ferulic acid, chlorogenic acid, procyanidin B1

Table S8. Chemical composition of bay laurel solid residue extracts analysed by HPLC. Major compounds (≥ 3 mg/g in any sample) are marked in **blue**.

**Bay laurel**	**w(mg/g)**														
	**Et-H_2O**					**Me-H_2O**					**Et-Me-H_2O**				
	**RAW**	**HD**	**HD-RE**	**HD-REXPC**	**HD-US**	**RAW**	**HD**	**HD-RE**	**HD-REXPC**	**HD-US**	**RAW**	**HD**	**HD-RE**	**HD-REXPC**	**HD-US**
**Epicatechin-hexoside**	3.19	N.D.	0.47	N.D.	0.85	N.D.	0.06	N.D.	0.06	N.D.	0.37	0.17	0.19	0.12	0.20
(-)-epicatechin*	2.92	0.45	0.20	0.21	0.25	0.63	0.34	0.33	0.23	0.33	7.24	0.43	0.26	0.31	0.28
(-)-epicatechin-3-*O*-gallate	1.13	0.12	N.D.	N.D.	0.33	N.D.	N.D.	N.D.	N.D.	N.D.	2.23	0.15	0.29	0.07	0.31
Procyanidin dimer II	1.32	N.D.	N.D.	N.D.	N.D.	1.19	0.37	N.D.	N.D.	N.D.	2.10	N.D.	N.D.	N.D.	N.D.
**Procyanidin trimer III**	8.17	4.84	2.26	2.12	3.82	6.08	2.28	2.75	1.45	5.96	27.81	5.70	6.22	2.83	8.67
Procyanidin tetramer II	0.35	0.44	N.D.	N.D.	0.21	0.29	0.86	N.D.	0.47	N.D.	1.03	0.53	N.D.	0.27	N.D.
Luteolin-6-*C*-glucoside	0.03	0.02	N.D.	N.D.	N.D.	0.01	0.02	N.D.	0.01	N.D.	0.05	0.03	0.01	0.01	0.01
Quercetin-3-*O*-rutinoside*	0.08	0.15	N.D.	0.07	N.D.	0.05	0.12	0.07	0.08	0.08	0.15	0.17	N.D.	0.10	N.D.
Quercetin-*O*-hexoside	0.29	0.30	0.06	0.14	0.11	0.22	0.21	N.D.	0.14	N.D.	0.59	0.33	0.09	0.22	0.11
Quercetin-*O*-pentoside	0.29	0.30	0.08	0.10	0.21	0.27	0.20	0.08	0.13	0.13	0.70	0.34	0.13	0.16	0.21
Quercetin-*O*-pentoside	0.35	N.D.	0.15	N.D.	0.30	0.28	N.D.	0.16	N.D.	0.20	0.64	N.D.	0.25	N.D.	0.30
Quercetin-*O*-rhamnoside	0.67	0.25	0.18	0.31	0.37	0.56	0.20	0.21	0.32	0.25	1.47	0.28	0.30	0.50	0.37
Apigenin-6-*C*-glucoside	0.10	0.06	N.D.	0.02	N.D.	0.06	0.05	N.D.	0.03	N.D.	0.20	0.07	0.03	0.03	0.03
Apigenin-8-*C*-glucoside	0.02	0.01	0.02	N.D.	0.03	0.01	0.01	0.02	N.D.	0.02	0.03	0.01	0.01	N.D.	0.01
Kaempferol-3-*O*-rutinoside*	0.22	0.10	0.05	0.04	0.09	0.17	0.08	0.06	0.05	0.07	0.48	0.11	0.08	0.06	0.09
Kaempferol-*O*-pentoside	0.77	0.62	0.27	0.04	0.52	0.62	0.44	0.29	0.04	0.35	1.61	0.70	0.45	0.07	0.52
Kaempferol-*O*-hexoside	0.68	0.42	0.16	0.17	0.34	0.51	0.29	0.17	0.17	0.20	1.69	0.47	0.30	0.28	0.04
Isorhamnetin-*O*-hexoside	0.07	0.14	0.04	0.02	0.08	0.08	0.06	0.05	0.02	0.05	0.16	0.10	0.07	0.04	0.08
Isorhamnetin-*O*-pentoside	0.09	0.03	0.03	N.D.	0.06	0.07	0.02	0.03	N.D.	0.04	0.24	0.03	0.06	N.D.	0.06
**TOTAL**	**20.74**	**8.25**	**3.97**	**3.24**	**7.57**	**11.10**	**5.61**	**4.22**	**3.20**	**7.68**	**48.79**	**9.62**	**8.74**	**5.07**	**11.29**

RAW - dry plant material subjected directly to the ultrasound assisted extraction, without hydrodistillation. HD - hydrodistillation without pre-treatment. HD-RE - hydrodistillation pre-treatment with reflux extraction. HD-REXPC - hydrodistillation pre-treatment with reflux extraction assisted with xylanase, pectinase and cellulase. HD-US – hydrodistillation pre-treatment with ultrasound extraction. N.D. - not detected. *Authentic standards used: (+)-catechin, (-)-epicatechin, apigenin, quercetin-3-O-glucoside, quercetin-3-O-rutinoside, kaempferol-3-*O*-rutinoside.

Table S9. Chemical composition of rosemary solid residue extracts analysed by HPLC. Major compounds (≥ 3 mg/g in any sample) are marked in **blue**.

Rosemary	w(mg/g)														
	Et-H_2O					Me-H_2O					Et-Me-H_2O				
	**RAW**	**HD**	**HD-RE**	**HD-REXPC**	**HD-US**	**RAW**	**HD**	**HD-RE**	**HD-REXPC**	**HD-US**	**RAW**	**HD**	**HD-RE**	**HD-REXPC**	**HD-US**
Gallocatehin	0.23	N.D.	0.35	N.D.	0.20	0.25	0.97	0.28	N.D.	0.19	0.26	N.D.	0.33	N.D.	0.18
Chlorogenic acid*	0.05	N.D.	0.02	N.D.	0.01	0.14	N.D.	0.02	N.D.	0.02	0.20	N.D.	0.02	N.D.	0.01
Caffeic acid*	0.01	0.23	0.05	0.47	0.04	N.D.	0.21	0.06	0.37	N.D.	N.D.	0.22	N.D.	0.49	N.D.
Syringic acid*	0.39	0.39	0.25	0.22	0.18	0.71	0.20	0.36	0.13	0.09	0.70	0.20	0.41	0.17	0.18
Ferulic acid*	0.02	N.D.	N.D.	N.D.	N.D.	N.D.	N.D.	N.D.	N.D.	N.D.	0.02	N.D.	0.03	N.D.	0.02
**Rosmarinic acid***	2.59	3.73	1.04	1.23	1.22	2.87	3.25	0.55	0.87	0.90	4.09	3.60	0.91	1.23	1.31
Hesperidin	0.14	N.D.	0.06	N.D.	0.07	0.15	N.D.	0.04	N.D.	0.05	0.21	N.D.	N.D.	N.D.	0.01
Apigenin*	0.16	0.18	0.19	0.32	0.23	0.65	0.28	0.12	0.19	0.18	0.83	0.38	0.19	0.31	0.23
Luteolin-7-*O*-glucuronide	0.05	N.D.	0.06	N.D.	0.07	0.07	N.D.	0.04	N.D.	0.06	0.10	N.D.	0.07	N.D.	0.07
Luteolin-3-*O*-glucuronid	0.05	0.13	N.D.	0.17	N.D.	N.D.	0.05	N.D.	0.06	N.D.	N.D.	0.02	N.D.	0.21	N.D.
**TOTAL**	**3.69**	**4.66**	**2.02**	**2.41**	**2.02**	**4.84**	**4.96**	**1.47**	**1.62**	**1.49**	**6.41**	**4.42**	**1.96**	**2.41**	**2.01**

RAW - dry plant material subjected directly to the ultrasound assisted extraction, without hydrodistillation. HD - hydrodistillation without pre-treatment. HD-RE - hydrodistillation pre-treatment with reflux extraction. HD-REXPC - hydrodistillation pre-treatment with reflux extraction assisted with xylanase, pectinase and cellulase. HD-US – hydrodistillation pre-treatment with ultrasound extraction. N.D. - not detected. *Authentic standards used: gallic acid, chlorogenic acid, caffeic acid, syringic acid, *p*--coumaric acid, ferulic acid, rosmarinic acid, apigenin, luteolin.

Supplementary Figures

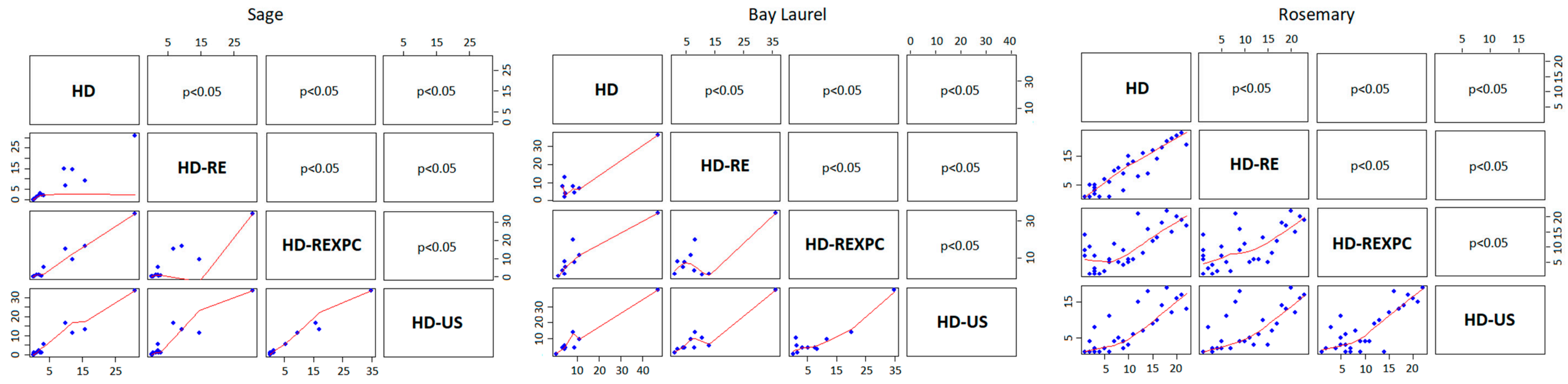

Figure S1. Scatter plot showing correlations between different pre-treatments (on the diagonal) regarding chemical composition of hydrolates. Significant *p*-values based on Spearman's rank test are shown above the diagonal, while bivariate scatter plots are shown below the diagonal. HD - hydrodistillation without pre-treatment. HD-RE - hydrodistillation pre-treatment with reflux extraction. HD-REXPC - hydrodistillation pre-treatment with reflux extraction assisted with xylanase, pectinase and cellulase. HD-US – hydrodistillation pre-treatment with ultrasound extraction.

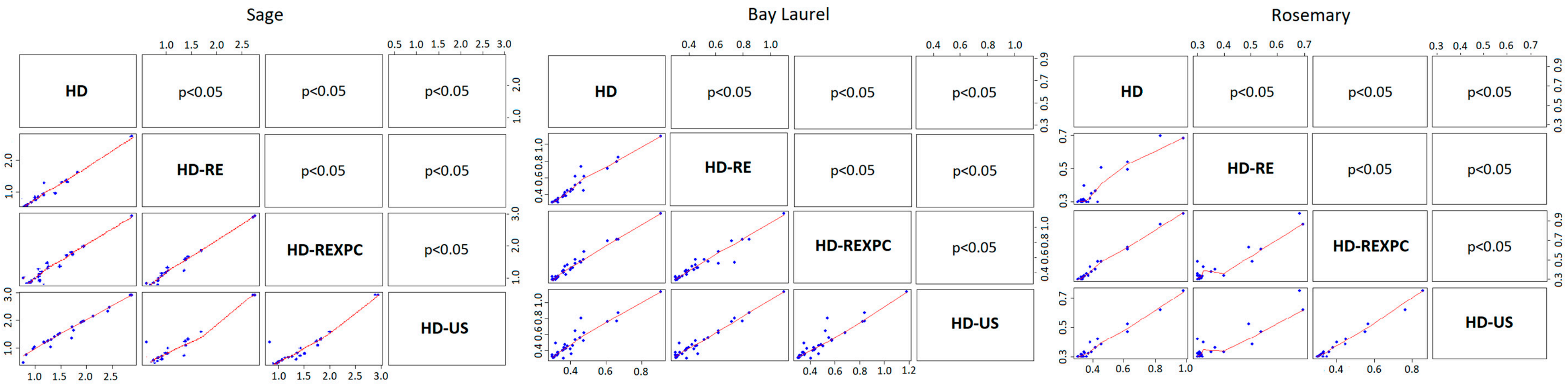

Figure S2. Scatter plot showing correlations between different pre-treatments (on the diagonal) regarding chemical composition of water residues. Significant *p*-values based on Spearman's rank test are shown above the diagonal, while bivariate scatter plots are shown below the diagonal. HD - hydrodistillation without pre-treatment. HD-RE - hydrodistillation pre-treatment with reflux extraction. HD-REXPC - hydrodistillation pre-treatment with reflux extraction assisted with xylanase, pectinase and cellulase. HD-US – hydrodistillation pre-treatment with ultrasound extraction.

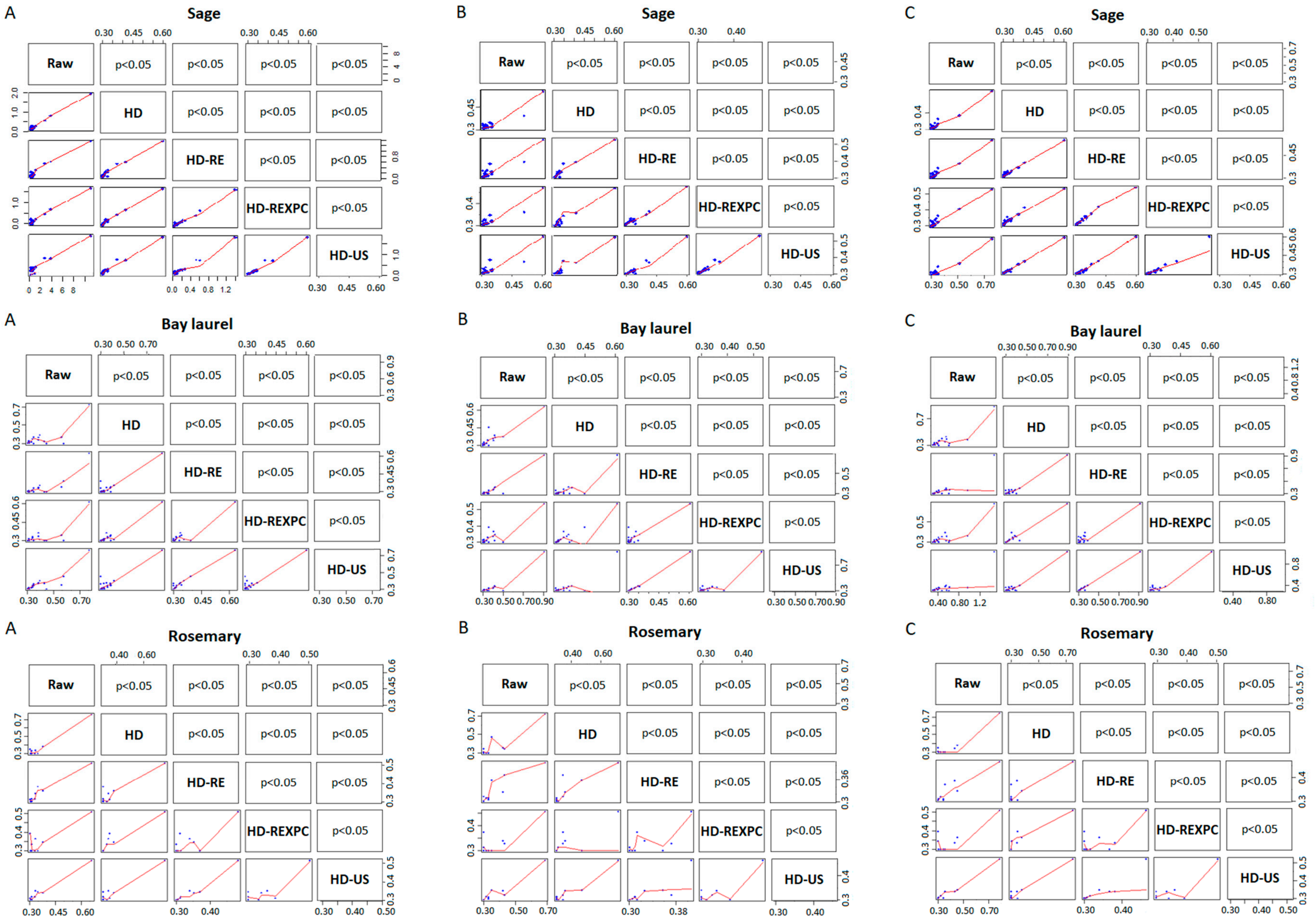
A
Sage
B
Sage
C
Sage
A
Bay laurel
B
Bay laurel
C
Bay laurel
A
Rosemary
B
Rosemary
C
Rosemary
Raw
HD
HD-RE
HD-REXPC
HD-US
p<0.05

Figure S3. Scatter plot showing correlations between different pre-treatments (on the diagonal) regarding chemical composition of solid residue extracts extracted with different solvents: A - ethanol (ethanol:water = 1:1), B - methanol (methanol:water = 1:1), C - ethanol-methanol (ethanol:methanol:water = 1:1:1). Significant p-values based on Spearman's rank test are shown above the diagonal, while bivariate scatter plots are shown below the diagonal. Raw - dry plant material subjected directly to the ultrasound assisted extraction, without hydrodistillation. HD - hydrodistillation without pre-treatment. HD-RE - hydrodistillation pre-treatment with reflux extraction. HD-REXPC - hydrodistillation pre-treatment with reflux extraction assisted with xylanase, pectinase and cellulase. HD-US – hydrodistillation pre-treatment with ultrasound extraction

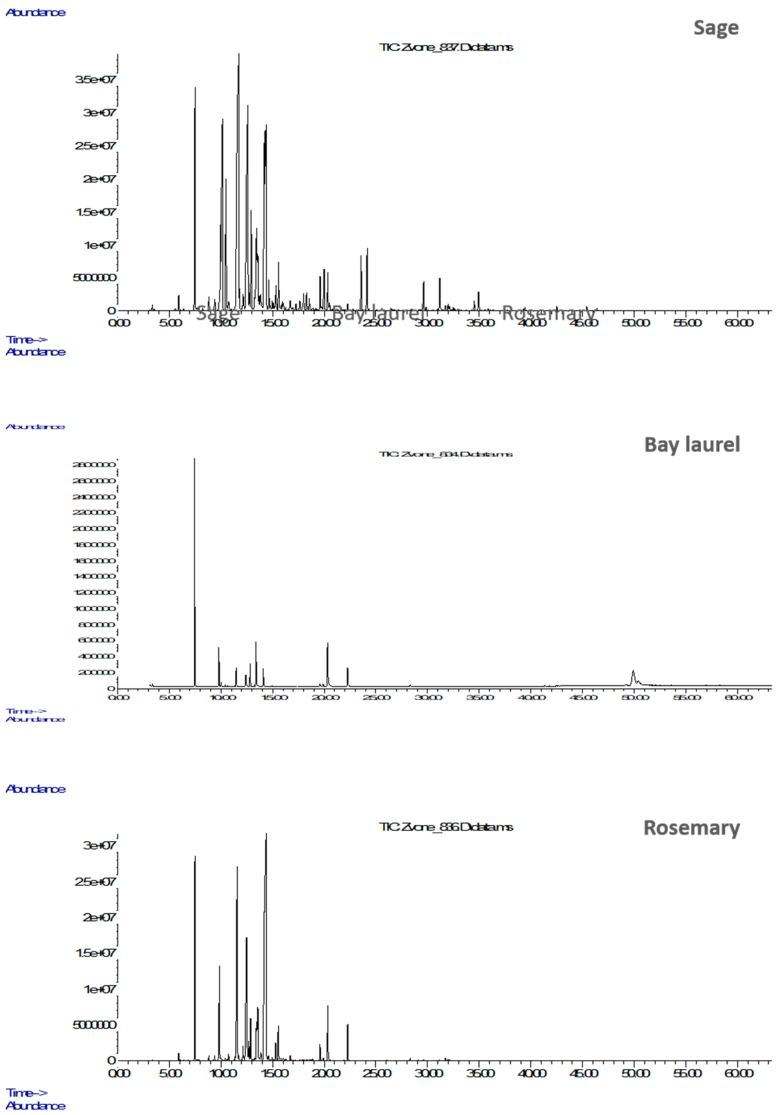

Figure S4. Total ion chromatograms of sage, bay laurel and rosemary hydrolates, obtained after hydrodistillation without pre-treatment (HD) and determined by GC-MS. Similar chemical profiles were obtained with different pre-treatments: HD-RE – hydrodistillation with reflux extraction pre-treatment, HD-US - hydrodistillation with ultrasound extraction pre-treatment, HD-REXPC – hydrodistillation with reflux extraction pre-treatment assisted with cell wall-degrading enzymes (xylanase, pectinase and cellulase).

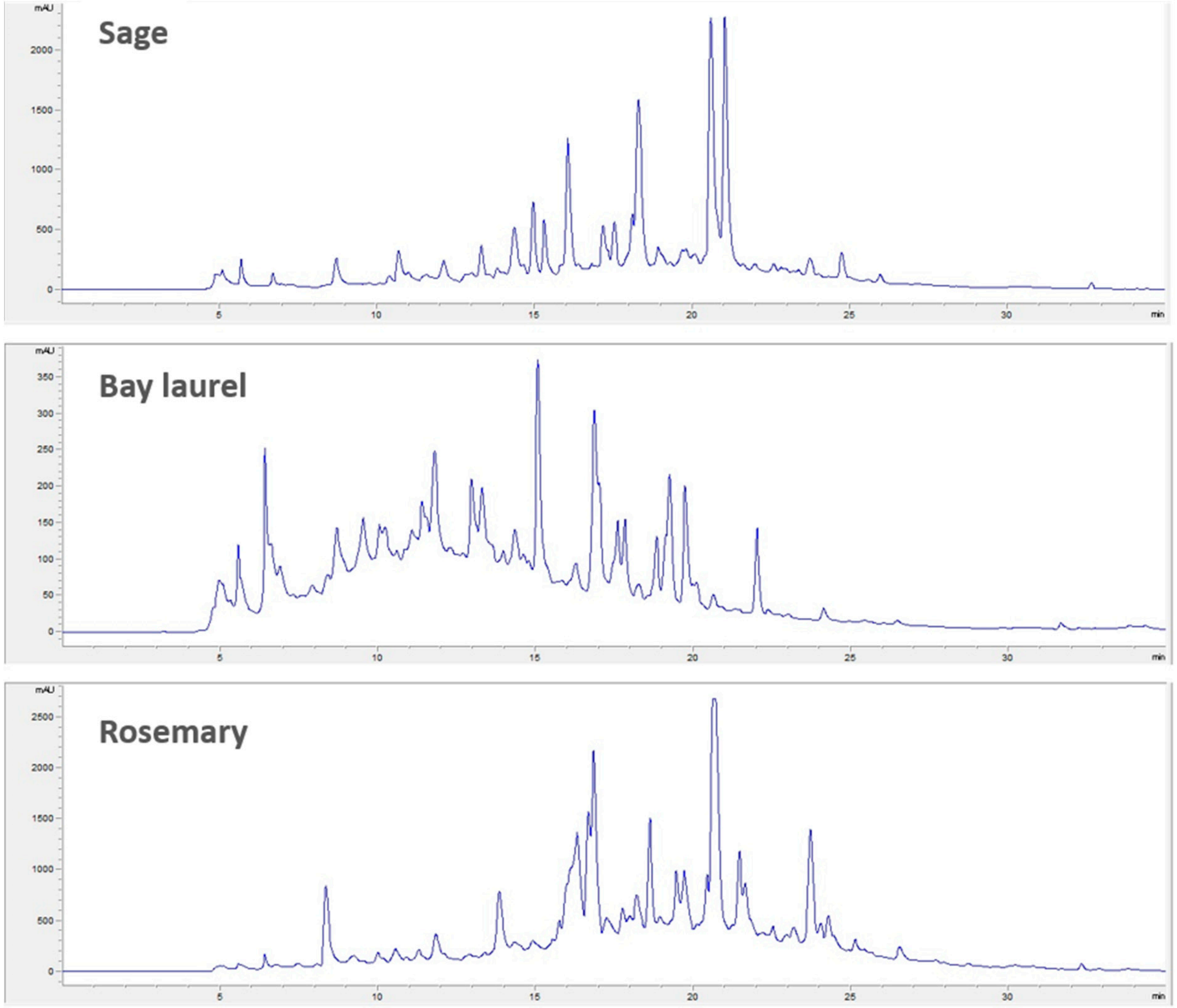

Figure S5. HPLC UV-VIS/PDA chromatograms of phenolic compounds obtained from sage, bay laurel and rosemary water residues and recorded at 278 nm. Similar chemical profiles were obtained with different pre-treatments: HD-RE – hydrodistillation with reflux extraction pre-treatment, HD-US - hydrodistillation with ultrasound extraction pre-treatment, HD-REXPC – hydrodistillation with reflux extraction pre-treatment assisted with cell wall-degrading enzymes (xylanase, pectinase and cellulase).

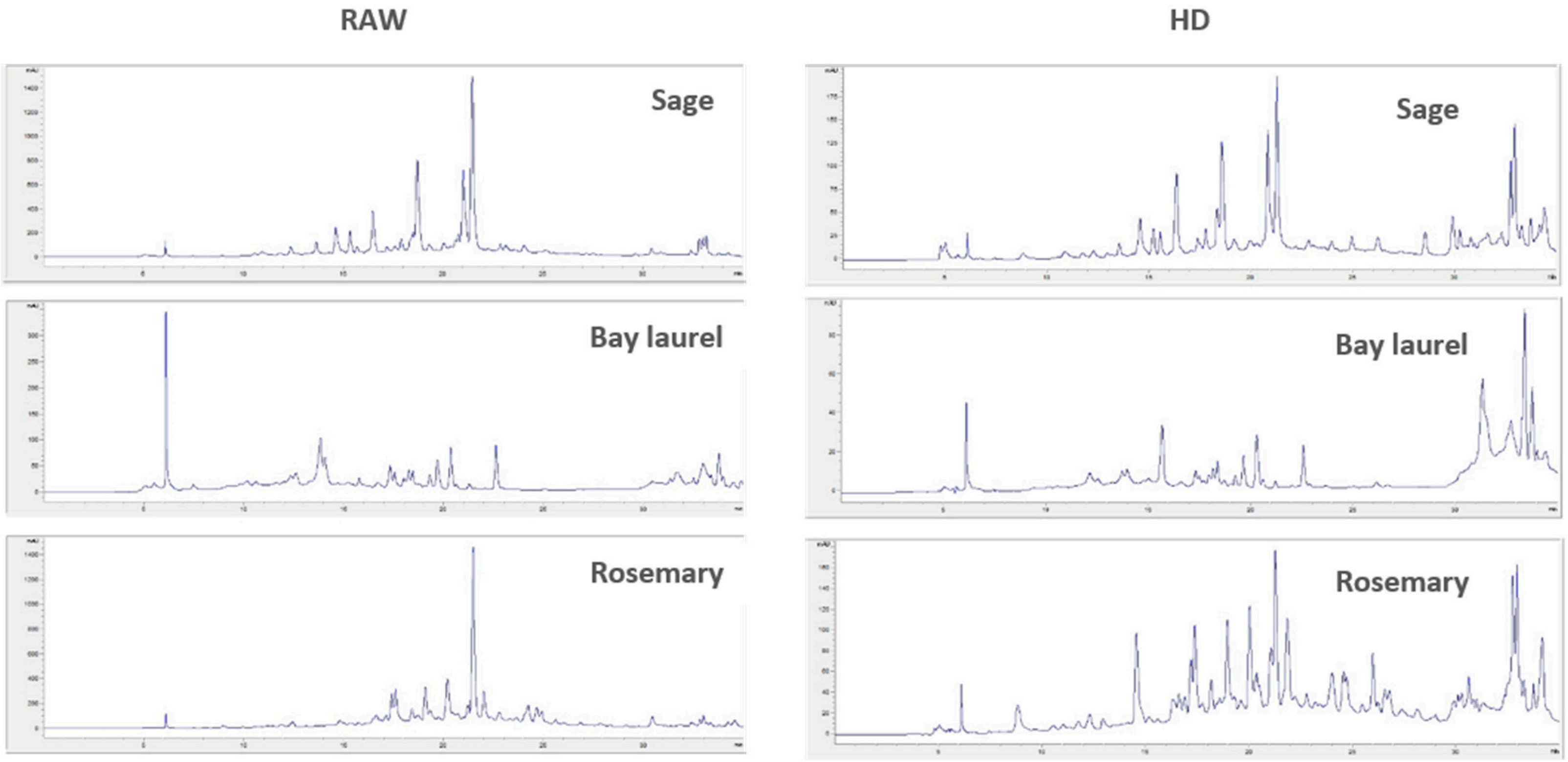

Figure S6. HPLC UV-VIS/PDA chromatograms of phenolic compounds obtained from sage, bay laurel and rosemary solid residues and recorded at 278 nm. Solid residues remaining after hydrodistillation (HD) were treated with ultrasound and Et-H_2O (ethanol : water = 1 : 1 v/v) as a solvent. Furthermore, hydrodistillation was omitted and dry plant material was subjected directly to ultrasound-assisted extraction with different solvents (RAW). Similar chemical profiles were obtained after different pre-treatments: HD-RE - reflux extraction, HD-REXPC - reflux extraction assisted with cell wall-degrading enzymes (xylanase, pectinase and cellulase), HD-US - ultrasound extraction; or with different solvents (Me-H_2O - methanol : water = 1 : 1 v/v; Et-Me-H_2O - ethanol : methanol : water = 1 : 1: 1 v/v/v).
